# Investigating the role of excipients on the physical stability of directly compressed tablets

**DOI:** 10.1016/j.ijpx.2021.100106

**Published:** 2021-12-09

**Authors:** Natalie Maclean, Ibrahim Khadra, James Mann, Helen Williams, Alexander Abbott, Heather Mead, Daniel Markl

**Affiliations:** a Strathclyde Institute of Pharmacy & Biomedical Sciences, University of Strathclyde, Glasgow, UK; b Oral Product Development, Pharmaceutical Technology & Development, Operations, AstraZeneca, Macclesfield, UK; c New Modalities and Parenteral Development, Pharmaceutical Technology & Development, Operations, AstraZeneca, Macclesfield, UK; d Future Continuous Manufacturing and Advanced Crystallisation (CMAC) Research Hub, University of Strathclyde, Glasgow, UK

**Keywords:** Physical stability, Accelerated stability, Sample storage, Disintegration mechanism

## Abstract

Stability studies are an integral part of the drug development process for any drug product. In addition to monitoring chemical degradation, the physical stability of a drug product must also be evaluated to ensure that the drug release and performance is not affected by storage. In this study, directly compressed tablets of 16 different formulations were exposed to an accelerated stability program to quantify changes in tablet breaking force, porosity, contact angle and disintegration time. Tablets were exposed to five different storage conditions from 37^∘^*C*/30% relative humidity (RH) to 70^∘^*C*/75%RH with testing after 2 and 4 weeks of storage. Each formulation contained two different fillers (47% *w*/w each), a disintegrant (5% *w*/w) and magnesium stearate (1% w/w). The results show that tablets stored at high humidity show increases in porosity and decreases in tensile strength, particularly if they contain a highly hygroscopic filler such as microcrystalline cellulose (MCC). For tablets stored at high temperature, the most commonly affected property was the tablet wettability, measured by sessile drop contact angle measurements. These results are considered in combination with the performance-controlling disintegration mechanism (Maclean et al., 2021) to identify the critical properties which influence the performance after storage.

## Graphical abstract

**Figure:**
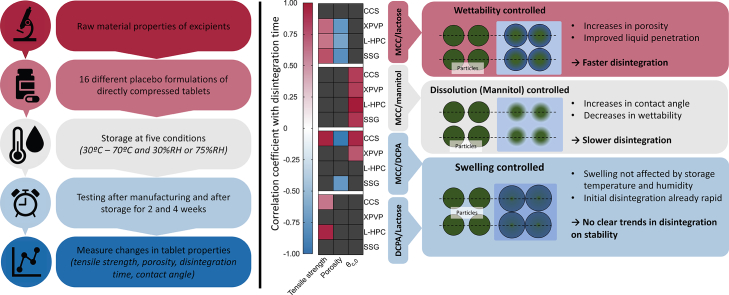

## 1 Introduction

Stability testing is a crucial step in the development of any drug product. The main focus of stability testing is on assessing the chemical degradation to ensure that the product remains safe throughout the duration of its shelf-life. Accelerated stability studies are routinely used to study chemical degradation, and this data is often used to extrapolate for long-term storage. In recent years, industrial interest in accelerated predictive techniques has grown rapidly (Qiu, 2018; Williams et al., 2017). In addition to chemical stability, the physical stability of pharmaceutical products must also be evaluated to ensure that the product performance is not affected by storage. The modelling and prediction of physical stability presents unique challenges, as physical changes do not always follow the Arrhenius behaviour which is used to predict chemical reactions. A recent survey by Williams et al. (2017) documented the properties currently being studied with risk-based predictive stability techniques in industry. Responses from 16 companies currently using these approaches, the most common applications were for studying chemical impurities and assays (13 and 11 companies, respectively), with only 3 companies using these tools for dissolution testing and only 1 respondent each applying these tools to study hardness or disintegration. By developing an improved understanding of the underlying mechanisms of physical change during storage, more appropriate predictive tools could be developed which better fit the needs of physical stability testing.

Many studies in the literature have discussed the physical stability of different drug products, however the wide variety of formulations (including the choice of both active pharmaceutical ingredient(s) and excipients), manufacturing processes, and study designs make it challenging to compare different studies.

Tablet excipients often account for a large portion of the total tablet mass, and so the excipients will typically influence the physical properties of the tablet. Several studies have investigated the effect of different excipients on the physical stability of tablets, including the impact of filler solubility (Molokhia et al., 1982; Gordon et al., 1993), formulation hygroscopicity (Gordon and Chowhan, 1990), and disintegrant efficacy (Quodbach and Kleinebudde, 2015).

The effect of filler solubility was investigated by Molokhia et al. (1982) and Gordon et al. (1993), who both attributed increases in tablet hardness after storage to the partial dissolution and recrystallisation of soluble fillers. The exception to this would be sorbitol-based tablets, which showed a decrease in hardness after storage (Molokhia et al., 1982). These changes were not found in tablet composed of mainly insoluble fillers. In addition to the change in hardness, Gordon et al. (1993) also found that tablets containing soluble fillers experienced a decrease in dissolution rate after storage under accelerated conditions. Filler hygroscopicity was also investigated to determine its effect on physical stability. Gordon and Chowhan (1990) found that tablets with a higher composite hygroscopicity show greater decreases in dissolution rate after storage at elevated humidity compared to those with non-hygroscopic excipients. In addition to factors like solubility and hygroscopicity, it has also been shown that the mode of deformation of a material can influence the physical stability, with differences in behaviour observed for brittle fracture fillers like dibasic calcium phosphate dihydrate (DCPD) and lactose monohydrate, compared to the plastically-deforming microcrystalline cellulose (MCC) (Sacchetti et al., 2017).

Several studies have also investigated the effects of storage on disintegrants (Gordon et al., 1993; Hersen-Delesalle et al., 2007; Quodbach and Kleinebudde, 2015; Hiew et al., 2016). Gordon et al. (1993) assessed changes in the physical properties of tablets containing croscarmellose sodium (CCS), crospovidone (XPVP) and low-substituted hydroxypropyl cellulose (L-HPC) as disintegrants. This study found that the behaviour on stability changed depending on the disintegrant used, for example, tablets containing CCS were more strongly affected by storage at 37^∘^*C*/80%RH despite still having a faster disintegration time than those containing XPVP or L-HPC. Hersen-Delesalle et al. (2007) found that a decrease in tensile strength with increased relative humidity was associated with the formation of cracks on the surface and internal structure of the tablets, likely due to premature activation of disintegrants as they absorb moisture from the air. Quodbach and Kleinebudde (2015) studied the effect of storage conditions on the water uptake and force development for tablets containing different disintegrants, finding that disintegration time increased after storage at high humidity for tablets containing two different grades of SSG. These changes are attributed to a plasticising effect on the polymer structure, resulting in the premature release of some of the shape recovery energy stored from compression. Li et al. (2004); Hiew et al. (2016) investigated the effect of relative humidity on dibasic calcium phosphate-based tablets with XPVP. After storage at 75% relative humidity (RH), the high moisture sorption of XPVP made the tablets become immeasurably soft and deformed. It was also shown by Sacchetti et al. (2017) that the moisture sorption properties of disintegrants cause volume expansion during storage, which in turn results in stress relaxation and prolonged disintegration times, particularly for tablets containing XPVP. This was further confirmed by Bauhuber et al. (2021), who showed that tablets containing XPVP showed the biggest change in disintegration time after storage at 40^∘^*C*/75%RH compared to those containing CCS or SSG.

For chemical stability, predictions are typically based on the reaction rates of chemical degradation pathways, for example using the Arrhenius equation. When considering physical stability changes, these often do not involve chemical changes, and so predicting the rate and extent of change can be challenging. In the literature, several studies have applied chemical stability techniques such as the Accelerated Stability Assessment Program (ASAP) or GSK's accelerated stability modelling (ASM) approach to study disintegration and dissolution changes on stability (Waterman, 2011; Li et al., 2004; Clancy et al., 2018). A study by Li et al. (2004), investigated the decrease in dissolution rate of benazepril hydrochloride tablets containing XPVP after storage at 40^∘^C/75%RH. In this study, the change in behaviour was attributed to disintegrant pre-activation, and a simple model was proposed to predict dissolution slowdown based on the moisture uptake of the tablets. A technique was also proposed by Scrivens (2019), which calculated an ‘acceleration factor’ (AF) which was used to correct the timescale of a dissolution profile of samples after storage to match it to the initial profile. The AF was found to decrease exponentially over time for each condition, and the calculation of the fitting parameters for this exponential allowed accurate predictions of the change in dissolution rate.

Previously, the formulations included in this study were classified as either dissolution controlled, wettability controlled or swelling controlled (Maclean et al., 2021). To do this, a workflow was developed which focused on the raw material and tablet properties, specifically the solubility and dissolution rate of the particles, as well as the porosity and wettability of the tablet. This classification process helped to identify the critical properties which influences the disintegration behaviour of the tablets. Following these classifications, the surface liquid-absorption and swelling processes were quantified using dynamic contact angle measurements for these formulations (Markl et al., 2021). The liquid-absorption and swelling results supported the proposed mechanisms of disintegration for each formulation, with the disintegration of MCC/mannitol and MCC/lactose tablets primarily being influenced by the liquid absorption, whilst disintegration of MCC/DCPA tablets were generally swelling-controlled, and tablets composed of DCPA/lactose tablets showed some influence from both the liquid-absorption and swelling behaviour.

The objective of this study is to assess the relationship between physical tablet properties including tensile strength, porosity, initial contact angle, and disintegration time with storage temperature and humidity during stability studies. This study compares the effect of storage temperature and humidity on 16 different directly compressed placebo formulations with four different filler-combinations and four commonly used disintegrants. The effect of exposure to accelerated stability conditions are also compared against the performance-controlling disintegration mechanism for each formulation prior to storage.

## 2 Materials and methods

### 2.1 Materials

Tablet excipients included microcrystalline cellulose (MCC) (Avicel®PH-102, FMC International), mannitol (Pearlitol®200 SD, Roquette), lactose (FastFlo®316, Foremost Farms USA) and dibasic calcium phosphate anhydrous (DCPA) (Anhydrous Emcompress, JRS Pharma) as fillers. The disintegrants croscarmellose sodium (CCS) (FMC International), crospovidone (XPVP) (Kollidon®CL, BASF), low-substituted hydroxypropyl cellulose (L-HPC) (LH-21, Shin-Etsu Chemical Co.) and sodium starch glycolate (SSG) (Primojel®, DFE Pharma) were used in the tablet formulations. Magnesium stearate (Mallinckrodt) was used as a lubricant. Magnesium chloride and sodium chloride (Sigma Aldrich) were used to make saturated salt solutions.

### 2.2 Dynamic vapor sorption (DVS)

Moisture sorption isotherms were collected for each of the fillers and disintegrants by DVS analysis at 37^∘^C, 50^∘^C, and 60^∘^C using the DVS Advantage (Surface Measurement Systems, London, UK). Samples of approximately 10 mg were analysed for each excipient. Prior to analysis, samples were conditioned at 0% relative humidity (RH). After conditioning, samples were analysed to measure the moisture content at 30% and 75%RH. At each humidity level, the change in mass was recorded once the balance reading had stabilised to less that 0.002% change in mass per minute. To calculate the theoretical liquid sorption for each formulation (*v*_mix_), a weighted average was calculated based on the weight fraction of each excipient (*c*_*i*_) and the individual moisture uptake values (*v*_*i*_) using

(1) $$v_{\mathrm{mix}}=\sum_{i=1}^{N}c_{i}\cdot v_{i}$$

with *N* = 3 as the number of excipients (excluding magnesium stearate) in the formulation used.

### 2.3 Tablet manufacture

Full details of the tablet manufacture can be found in Maclean et al. (2021). Briefly, 16 different direct compression formulations were manufactured. Each batch contained a combination of two fillers (47% *w*/w each), a disintegrant (5% *w*/w) and magnesium stearate as a lubricant (1% w/w). The formulations used are shown in Table 1.

**Table 1:** Tablet formulations.

Filler 1	Filler 2	Disintegrant
MCC	Mannitol	CCS
MCC	Mannitol	XPVP
MCC	Mannitol	L-HPC
MCC	Mannitol	SSG
MCC	Lactose	CCS
MCC	Lactose	XPVP
MCC	Lactose	L-HPC
MCC	Lactose	SSG
MCC	DCPA	CCS
MCC	DCPA	XPVP
MCC	DCPA	L-HPC
MCC	DCPA	SSG
DCPA	Lactose	CCS
DCPA	Lactose	XPVP
DCPA	Lactose	L-HPC
DCPA	Lactose	SSG

300 g of each blend was prepared by mixing the fillers and disintegrant for 20 min using a Pharmatech AB-015 bin blender at 20 rpm blend speed and 200 rpm agitator speed, the magnesium stearate was then added and the mixture was blended for a further 5 min using the same speed settings.

Tablets were compressed using an automated single-punch tablet press at 10 kN for tablets containing MCC/mannitol, MCC/lactose and MCC/DCPA and 16 kN for tablets containing DCPA/lactose.

### 2.4 Sample storage

For the stability studies, tablets were stored at five different temperature and humidity conditions as shown in Table 2. Tablets were placed in airtight glass jars containing an open vial saturated salt solution to control the relative humidity (magnesium chloride for 30%RH and sodium chloride for 75%RH, as suggested by Greenspan (1977)). The jars were then stored in a temperature-controlled room (37^∘^C and 50^∘^C) or oven (70^∘^C). Samples were removed from the oven for testing after 2 and 4 weeks. At each timepoint, jars were opened and allowed to equilibrate to ambient temperature and humidity of approximately 20-23^∘^C and 50–60%RH for a minimum of 3 days prior to testing.

**Table 2:** Accelerated stability storage conditions.

Temperature (^∘^C)	Humidity (%RH)	Timepoints (weeks)
37	30	0, 2, 4
37	75	0, 2, 4
50	75	0, 2, 4
70	30	0, 2, 4
70	75	0, 2, 4

### 2.5 Characterisation of tablets

#### 2.5.1 Weight, dimensions and tensile strength

The weight of each tablet was measured to the nearest 0.1 mg using an analytical balance. The diameter and thickness of each tablet were measured using a set of digital callipers and reported to the nearest 0.1 mm.

The hardness of each tablet was measured using a hardness tester (Copley TBF 1000, Copley Scientific Ltd., Nottingham, UK). The tensile strength, *σ*_*t*_, of tablets was calculated using the tablet breaking force, *F*, diameter, *d*, and height, *h* (Fell and Newton (1970):

(2) $$\sigma_{t}=\frac{2\cdot F}{\pi \cdot d\cdot h}.$$

The weight, dimensions and tensile strength are reported as the mean of 10 tablets per timepoint and condition for each formulation.

#### 2.5.2 Porosity

The porosity, *ε*, of tablets was calculated using the measured weight, *m*, dimensions and the true density, ρ_*t*, mix_, of the formulation. ρ_*t*, mix_ was calculated as the weighted harmonic mean considering the true density, ρ_*t*, *i*_, and the weight fraction, *c*_*i*_, of each excipient (Sun et al., 2018):

(3) $$\varrho_{t,\mathrm{mix}}={\left(\sum_{i=1}^{N}\frac{c_{i}}{\varrho_{t,i}}\right)}^{-1}$$

with *N* = 4 as the number of different excipients in the formulation. The porosity can then be calculated using

(4) $$\varepsilon =1-\frac{\frac{m}{\pi \cdot {\left(d/2\right)}^{2}\cdot h}}{\varrho_{t,\mathrm{mix}}}$$

The porosity was calculated for the 10 tablets used to measure weight, dimensions and tensile strength for each formulation at each timepoint and condition.

#### 2.5.3 Dynamic contact angle

Dynamic contact angle measurements were taken using a drop shape analyser (Krüss DSA30, Krüss GmbH, Hamburg, Germany). Video recordings were taken at a rate of 30 frames per second as a single droplet of MilliQ® Ultra-pure water was dispensed on to the surface of the tablet. The video files were analysed using MATLAB (R2019a, MathWorks, Massachusetts, USA) to determine the contact angle between the droplet and the tablet surface at each frame in the recording.

Two tablets were tested for each formulation. Using the data collected within the first second of contact with the liquid droplet for each pair of replicates, a two-phase exponential decay model (Eq. (5)) was fitted to the data using GraphPad Prism 9 (version 9.0.2, GraphPad Software LLC, San Diego).

(5) $$\begin{array}{c} \theta_{c}\left(t\right)=\theta_{c,p}+s_{f}e^{-k_{f}\cdot t}+s_{s}e^{-k_{s}\cdot t}\\ s_{f}=\theta_{c,0}\cdot x_{\mathrm{fs}}\\ s_{s}=\theta_{c,0}\left(1-x_{\mathrm{fs}}\right) \end{array}$$

*θ*_*c*, 0_ and *θ*_*c*, *p*_ are the contact angles at initial and infinite time, respectively. *k*_*f*_ and *k*_*s*_ are the rate constants for the fast and slow phases, respectively. The fraction of time dominated by the fast phase of the reaction is described as *x*_*fs*_.

The data collected during the dynamic contact angle measurements were also modelled to provide information about the liquid absorption and swelling kinetics, which is discussed by Markl et al. (2021) for tablets of each filler combination with CCS.

#### 2.5.4 Disintegration time

The disintegration time of tablets was measured using a Copley DTG 2000 Disintegration Tester (Copley Scientific Ltd., Nottingham, UK). Tablets were disintegrated in 800 mL of distilled water at 37^∘^*C*. Disintegration time was measured in seconds for 6 tablets per formulation. The mean and standard deviation of these 6 tablets is reported.

#### 2.5.5 Statistical analysis

Pearson correlation coefficients were calculated using GraphPad Prism 9 (version 9.0.2, GraphPad Software LLC, San Diego) to identify correlations between physical tablet properties and the storage conditions. Correlations were considered significant for *p* < 0.05.

## 3 Results

### 3.1 Moisture sorption

**Fig. 1:**
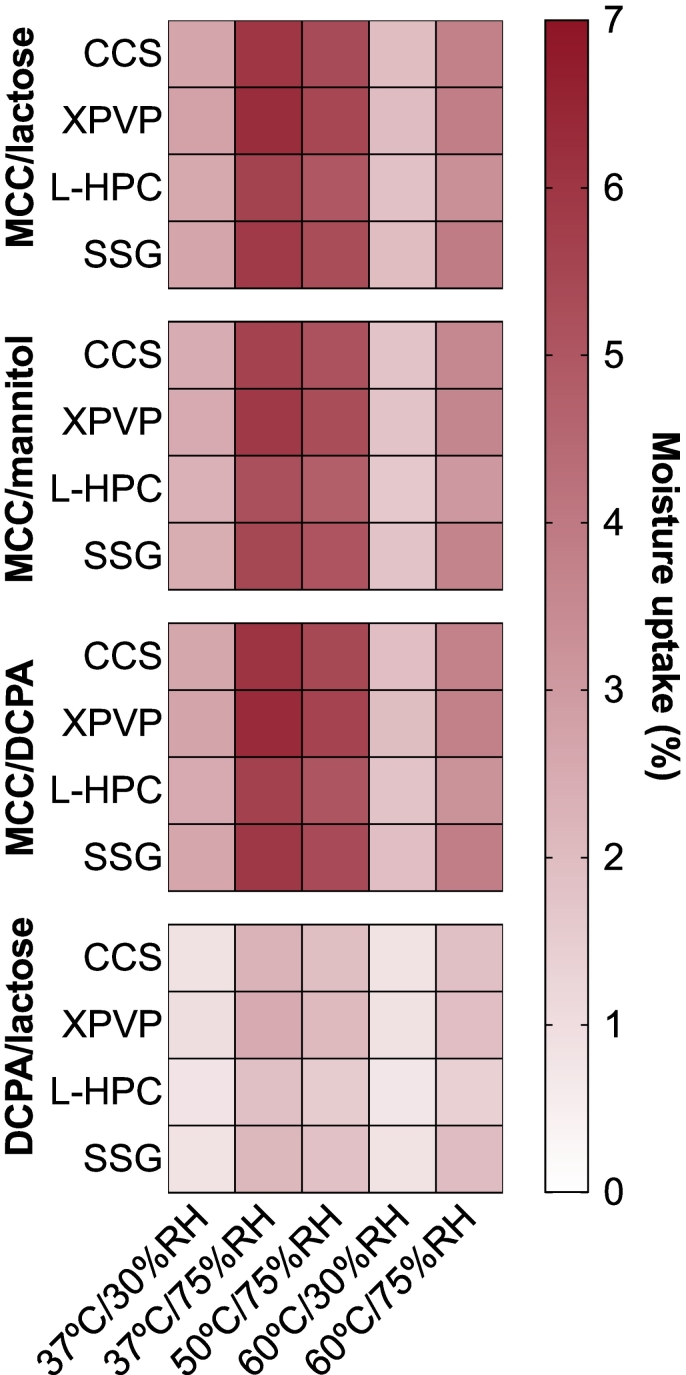
The moisture uptake for each formulation (%), based on the individual moisture sorption of each excipient.

The moisture sorption of each excipient was measured to simulate the conditions used in this study, with the exception of 70^∘^C conditions. Due to temperature limits of the instrument, 60^∘^C was used to replace 70^∘^C. The results of the moisture uptake are shown in Fig 1. Formulations containing MCC (MCC/mannitol, MCC/lactose, and MCC/DCPA) experienced increased moisture uptake at high humidity conditions, whilst 30%RH conditions showed smaller increases in moisture uptake. DCPA/lactose-based tablets only showed small changes in moisture uptake for all conditions. The individual moisture sorption isotherms for each excipient at 25^∘^*C* were shown in Maclean et al. (2021), and these profiles indicate that the main excipients contributing toward moisture uptake is MCC and the disintegrants.

### 3.2 Tensile strength

**Fig. 2:**
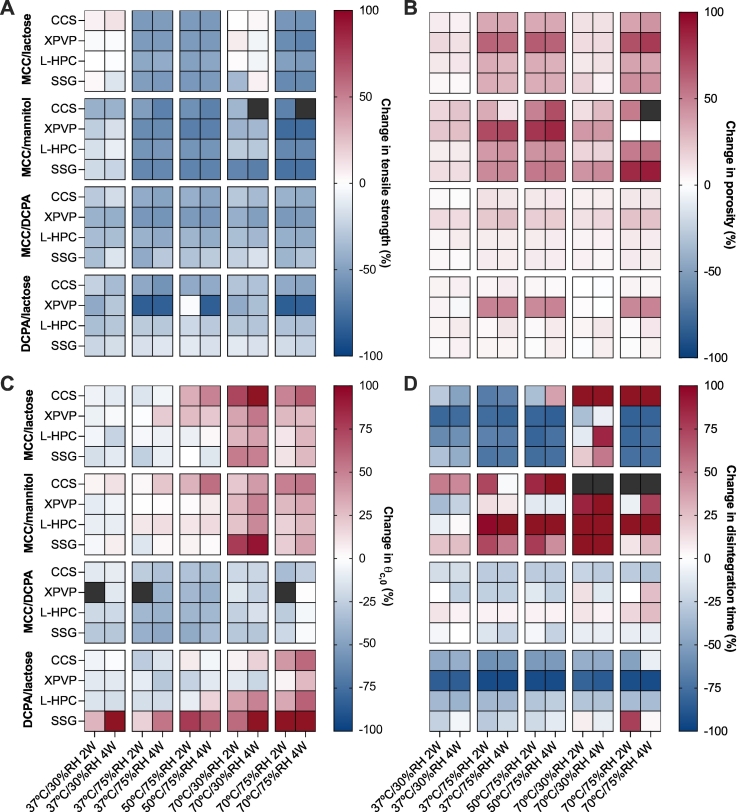
The change in (A) tensile strength, (B) porosity, (C) *θ*_*c*, 0_, and (D) disintegration time for all batches after storage under accelerated conditions. Grey data points represent points where no data is available.

The change in tensile strength for each batch during the stability study is shown in Fig. 2A. For tablets composed of MCC/lactose, there is little change in tensile strength for samples stored at low humidity. However, at high humidity, there is a decrease of around 40–60%. Tablets containing MCC/mannitol generally decreased in tensile strength after storage at all conditions. MCC/mannitol-based tablets with XPVP, L-HPC and SSG as the disintegrant experienced a slightly larger decrease in tensile strength after storage at high humidity compared to storage at low humidity. DCPA-based tablets (MCC/DCPA and DCPA/lactose) also decreased in tensile strength after storage. The changes for these batches were generally smaller than those shown by other filler combinations with the exception of DCPA/lactose tablets containing XPVP, which decreased the most out of all batches tested. The changes in tensile strength for DCPA-based tablets were also more uniform across different conditions compared to the other filler combinations. The full data sets for tensile strength during storage are shown in Figs. S1 - S4 in the Supporting Information. In most cases, changes in tensile strength occurred between the 0 and 2 week timepoints, after which point the tensile strength seemed to have reached a plateau.

### 3.3 Porosity

The changes in porosity during storage for all batches are shown in Fig. 2B. Tablets composed of MCC/mannitol and MCC/lactose show a slight increase in porosity after storage at 30%RH and a larger increase for tablets stored at 75%RH. At high humidity conditions, hygroscopic excipients such as MCC and the disintegrants can absorb moisture from the air (as shown by the moisture uptake data in Fig 1), resulting in particle expansion (often termed ‘disintegrant pre-activation’) (Berardi et al., 2021). After removal from storage, the absorbed moisture will subsequently be lost and enlarged or swollen particles will shrink to their original size, however, the changes to the microstructure caused by this particle expansion will be irreversible.

For MCC/mannitol and MCC/lactose the initial porosity (around 13%) is much lower than that of the MCC/DCPA and DCPA/lactose batches (around 23% and 19%, respectively) (Maclean et al., 2021). In the case of DCPA-based tablets, it was previously demonstrated that increases in porosity did not significant affect the disintegration time due to the swelling-controlled disintegration mechanism of this batch (Maclean et al., 2021). The disintegration time of swelling-controlled tablets is less influenced by changes in the tablet microstructure than the wettability- or dissolution-controlled tablets, and so the increase in porosity associated with disintegrant pre-activation during storage is less significant for the DCPA-based batches.

Within each filler combination, the largest increases in porosity are found with the batch containing XPVP as a disintegrant. This could indicate that the different mechanism of action of the disintegrants influences the stability of the formulation. Alternatively, the larger changes observed for these batches could be attributed to the higher moisture sorption capacity of XPVP (Maclean et al., 2021), or the premature release of energy by strain recovery for this disintegrant.

Profiles of tablet porosity during storage are shown in Figs. S5 - S8 in the Supporting Information. Similarly to the tensile strength profiles, these figures demonstrate that the porosity of the tablets generally exhibits the most change between 0 and 2 weeks, then remaining relatively constant between 2 and 4 weeks.

### 3.4 Contact angle

The changes in initial contact angle for each formulation during the stability study is shown in Fig. 2C. The individual plots of *θ*_*c*, 0_ at each timepoint are shown in Fig. S9-S12 in the Supporting Information. The changes in contact angle appear to vary within each of the filler combinations used in this study.

MCC/mannitol- and MCC/lactose-based tablets generally show large increases in *θ*_*c*, 0_ after storage at high temperature, particularly at 70^∘^C. For tablet stored at 37^∘^C there are only small changes in *θ*_*c*, 0_. These results suggest that increasing temperature results in decreased wettability for these batches.

When MCC/DCPA is used as the filler combination instead, *θ*_*c*, 0_ generally decreased for all batches by around 20–40% of the initial value. For these tablets, the change in *θ*_*c*, 0_ occurs between the initial and 2 week timepoint, and then remains constant between the 2 and 4 week timepoint.

The behaviour of tablets composed of DCPA/lactose vary depending on the disintegrant used. A large increase in *θ*_*c*, 0_ can be observed for tablets containing SSG across all timepoints and conditions. Tablets with CCS and L-HPC as the disintegrant only show a slight increase in *θ*_*c*, 0_ at high temperatures. When XPVP was used as a disintegrant, *θ*_*c*, 0_ generally decreased slightly for all conditions and timepoints except for tablets stored at 70^∘^*C*/75%RH for 4 weeks.

### 3.5 Disintegration time

The changes in disintegration time on stability are shown in Fig. 2D. The full stability profiles for tablet disintegration time are found in Figs. S13-S16 in the Supporting Information.

MCC/lactose-based tablets generally showed a decrease in disintegration time for tablets stored at every condition except 70^∘^*C*/30%RH for tablets with CCS, L-HPC and SSG as the disintegrant. For the batch which used CCS as disintegrant, there was also an increase in disintegration time at 70^∘^*C*/75%RH.

When MCC/mannitol was used as the filler combination, there were large increases in disintegration time for most storage conditions. For tablets containing CCS and SSG, the disintegration time increased for every storage condition. In particular, tablets with CCS as the disintegrant, storage at 70^∘^*C* resulted in tablets which did not fully disintegrate even after 20 min, and instead formed a gel-like consistency. The disintegration time of tablets containing L-HPC as the disintegrant increased at all conditions except 37^∘^*C*/30%RH. The MCC/mannitol batch least affected by storage was the batch containing XPVP as the disintegrant, which experienced an increased disintegration time at 70^∘^*C*/30%RH (2 weeks and 4 weeks) and 70^∘^*C*/75%RH (4 weeks).

For tablets containing DCPA, changes in disintegration time were generally much smaller. The exception is tablets containing DCPA/lactose with XPVP, which showed a consistent decrease in disintegration time at all conditions. It should also be noted that for tablets containing MCC/DCPA and DCPA/lactose, the initial disintegration time was already short (< 60 s (Maclean et al., 2021)), and so even moderate relative changes in disintegration time are only a few seconds in real-time.

### 3.6 Effects of storage time

The changes in tensile strength, porosity, initial contact angle and disintegration time over time can be found in the Supporting Information. In most cases, changes in the physical properties of the tablets occur within the first 2 weeks of storage, and then there is little change between the 2 and 4 week timepoints. This suggests that temperature- or humidity-induced changes are generally occuring within the 2 weeks of storage before reaching a constant state, which could mean that the changes are only dependent on storage conditions and would not occur during long-term storage at ambient conditions. There are some exceptions to this, for example, the disintegration time of MCC/lactose/L-HPC tablets stored at 70^∘^*C*/30%RH, and MCC/mannitol/XPVP tablets stored at 70^∘^*C*75%RH, which demonstrated little change in the first 2 weeks and a significant increase between 2 and 4 weeks. Conversely, the disintegration times of MCC/mannitol/CCS tablets stored at 37^∘^*C*/75%RH increased within the first 2 weeks, before returning to the initial disintegration time after 4 weeks.

### 3.7 Correlation between temperature, humidity and physical tablet properties

**Fig. 3:**
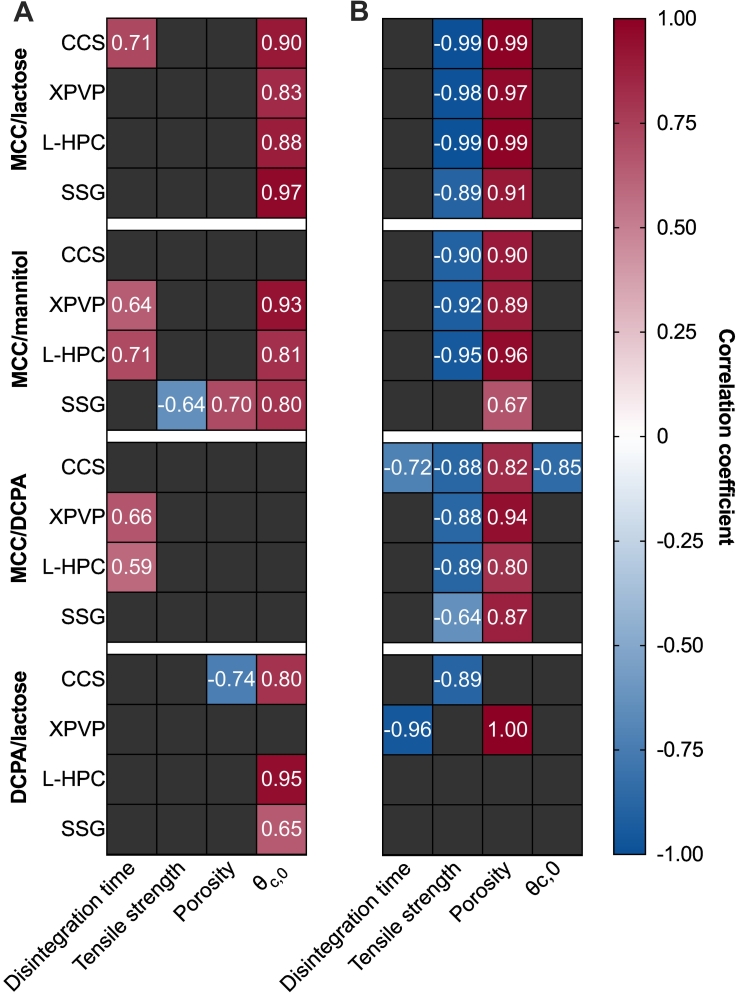
Pearson correlation coefficient between (A) storage temperature and (B) storage humidity with physical tablet properties. Only significant correlations (*p* < 0.05) are shown.

The correlation coefficients for physical tablet properties with storage temperature and humidity are depicted in Fig. 3A and B, respectively. Only statistically significant correlations (*p* ⩾ 0.05) are shown here.

There is a strong correlation (⩾0.80) between storage temperature and dynamic contact angle for all MCC/lactose-based tablets, most MCC/mannitol-based tablets (except those containing CCS) and most DCPA/lactose-based tablets (except those containing XPVP). This correlation suggests that for those batches, an increase in temperature results in an increase in *θ*_*c*, 0_, indicating a decrease in wettability. The wettability of a surface is influenced by three factors – porosity, surface roughness and surface energy. Comparing the porosity and contact angle, it seems that porosity changes are mainly based on humidity, whereas the contact angle is changing in response to storage temperature instead (Fig 2). When we consider surface roughness, we can assume that the surface roughness is not decreasing during storage, however, it may increase slightly as a result of premature swelling of some particles and the changes in porosity. If surface roughness increases, the contact angle for these samples would be expected to decrease (Wenzel, 1936). This suggests that the only property left which could be influencing the contact angle is a change in surface energy, or possibly surface chemistry, which is driven by high temperatures.

In some cases, this decrease in wettability is also reflected by an increase in disintegration time (MCC/lactose-based tablets with CCS and MCC/mannitol-based tablets with XPVP and L-HPC) with increasing storage temperature, however in most cases only the *θ*_*c*, 0_ correlates with temperature. For all MCC/DCPA-based tables, there is no correlation between porosity, tensile strength and *θ*_*c*, 0_ with storage temperature. For tablets containing XPVP and L-HPC with MCC/DCPA, the disintegration time positively correlates with the storage temperature – indicating that an increase in storage temperature results in slower disintegration. This correlation between disintegration time and storage temperature can also be observed for a few other formulations, specifically MCC/mannitol-based tablets with XPVP and L-HPC, and MCC/lactose tablets with CCS.

Correlation coefficients indicate that for most formulations (particularly those containing MCC), tensile strength and porosity have strong correlations with relative humidity (Fig. 3B). The positive correlation with porosity suggests that when stored at high relative humidity, the porosity of tablets would increase, whilst tensile strength would generally decrease (as shown in Fig. 2B and 2A, respectively). This can be explained by considering the effect of premature swelling of excipients such as MCC or the disintegrants. During storage at elevated humidity conditions, water is absorbed by hygroscopic, swelling particles, and after removal from storage this additional water is gradually lost. As water is absorbed, the pore space expands and remains permanently altered even after the loss of moisture. As the pore space expands, the bonds between the particles are weakened, resulting in the decreased tensile strength observed in most batches. These correlations are strongest in batches containing MCC, in which both MCC and the disintegrant may undergo premature swelling. For tablets containing DCPA/lactose, these correlations are generally not significant, with the exception of porosity for tablets containing XPVP and tensile strength for tablets containing CCS tablets. For these batches, premature swelling only occurs for the disintegrant particles as DCPA and lactose are not capable of swelling.

### 3.8 Correlation between physical tablet properties and disintegration time

**Fig. 4:**
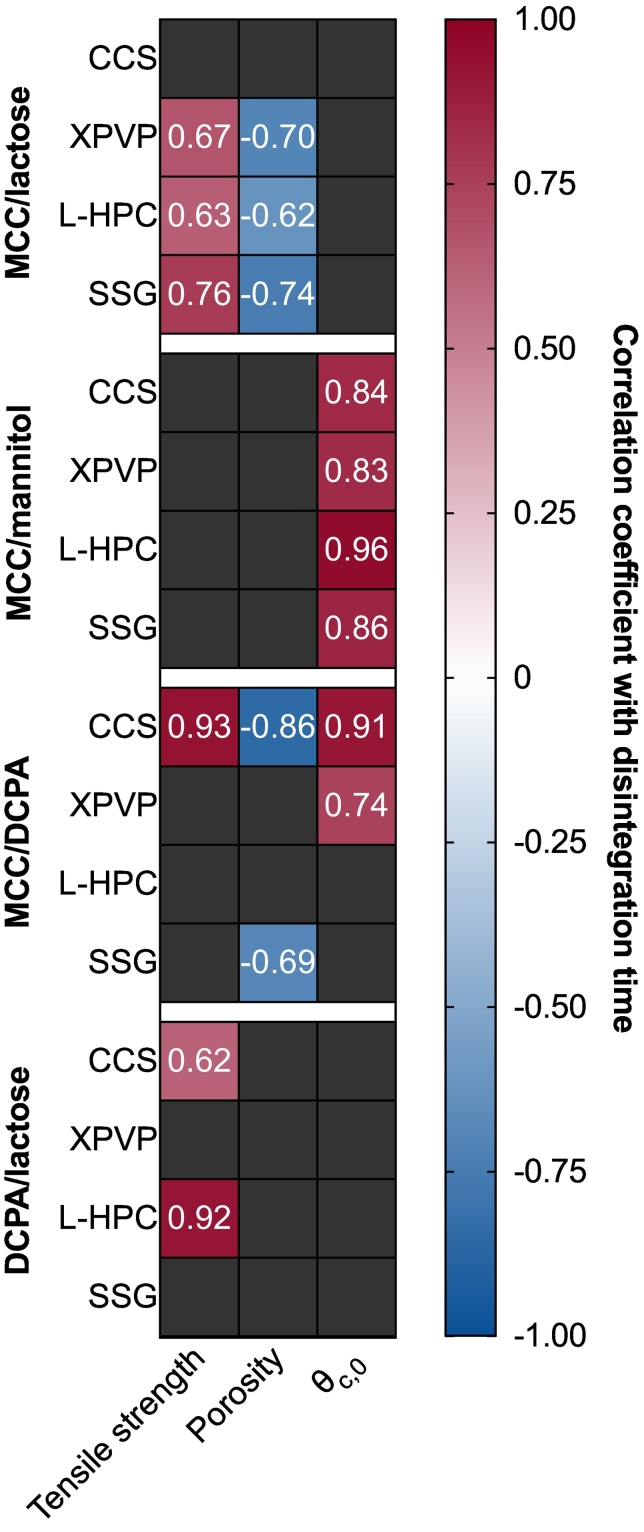
Pearson correlation coefficient between the disintegration time and the tensile strength, porosity and initial contact angle of all tablet during storage.

The correlation coefficient between disintegration time and the physical tablet properties (porosity, tensile strength and *θ*_*c*, 0_) are denoted in Fig. 4. Tablets composed of MCC/lactose (with the exception of CCS) show a significant correlation between both porosity and tensile strength with disintegration time. The correlation between porosity and disintegration time is negative, indicating that an increase in porosity is associated with a decrease in disintegration time. For tensile strength, there is a positive correlation with disintegration time. This suggests that as tensile strength decreases, disintegration time also decreases. These results can be explained by considering the absorption of moisture and subsequent swelling described previously. The disintegration performance of MCC/lactose was categorised as wettability-limited (Maclean et al., 2021). In this case, the increase in porosity can result in faster liquid penetration, which improves the rate of wetting and in turn facilitates faster disintegration.

The disintegration times of MCC/mannitol-based tablets show a strong positive correlation (> 0.8) with *θ*_*c*, 0_ for each disintegrant. The disintegration mechanism of MCC/mannitol tablets were previously described as dissolution controlled (Maclean et al., 2021). For these tablets (except those containing CCS), the contact angle is shown to increase with increased storage humidity (Fig. 3B). This increase in contact angle is also associated with an increase in disintegration time. The increase in *θ*_*c*, 0_ indicates a decrease in wettability, which could contribute toward slower wetting of mannitol particles, and thus, slower dissolution of mannitol from the tablet matrix. As this was previously determined to be the performance-limiting mechanism for disintegration, it follows that disintegration slows down after storage.

DCPA-based tablets (MCC/DCPA and DCPA/lactose) were previously classified as swelling-controlled, based primarily on the rapid disintegration times and high porosity. For these batches, initial disintegration time was rapid and changes during storage were generally small. For this reason, correlations between disintegration time and the physical tablet properties are generally low.

## 4 Discussion

### 4.1 MCC/lactose tablets

MCC/lactose-based tablets were previously classified as wettability controlled (Maclean et al., 2021). For these tablets at the initial timepoint, disintegration is slow due to a combination of the low porosity of the tablets and the slow dissolution of lactose from the tablet matrix. Changes in *θ*_*c*, 0_ of MCC/lactose-based tablets do not significantly correlate with disintegration time, suggesting that although *θ*_*c*, 0_ is shown to change based on the storage conditions, this does not necessarily influence the disintegration time for these tablets. Instead, increases in porosity and decreases in tensile strength are the key changes which result in decreases in disintegration time. This can be attributed to improved wettability due to increases porosity, and a lower force (due to reduced interparticle bonding strength) being required to break the tablet apart. This confirms the classification of MCC/lactose-based tablets as wettability controlled, as the disintegration at initial was limited by the low porosity (low liquid uptake) and the slow dissolution of lactose.

After storage at high temperature *θ*_*c*, 0_ generally increases for these tablets, indicated by the strong correlation coefficient between storage temperature and *θ*_*c*, 0_ (Fig. 3A). Fig. 3B shows that there is also strong correlations between the relative humidity during storage and porosity with tensile strength. After storage at elevated humidity, tablets expand due to premature swelling of MCC and the disintegrants, resulting in increased porosity and lower tensile strength.

### 4.2 MCC/mannitol tablets

Previously, these MCC/mannitol tablets were classified as dissolution controlled as the disintegration was primarily driven by the rapid dissolution of mannitol from the tablet matrix (Maclean et al., 2021). This resulted in an increase in pore space, increased liquid penetration and thus rapid disintegration. The results of this study demonstrate that the disintegration time of these tablets is primarily correlated with *θ*_*c*, 0_, which indicates that changes in disintegration time on stability can be associated primarily with changes to the wettability of the tablet. If the wettability of the tablet decreases during storage – specifically accelerated temperatures – then the dissolution of mannitol could be slowed due to slower wetting of the mannitol particles (Lu et al., 2014).

### 4.3 DCPA-based tablets

The correlation coefficients of tablets composed of MCC/DCPA or DCPA/lactose must be interpreted with caution. For these batches, the initial disintegration time is so rapid (< 30 s for MCC/DCPA tablets and < 60 s for DCPA/lactose tablets) that although changes in disintegration time can be seen in Fig. 2D, these changes are actually only a few seconds for each condition. Due to the relatively low change in disintegration time, it is difficult to draw conclusions based on the apparent correlations shown in Fig 4.

However, for these tablets we observe that there are very small changes in the porosity, presumably as the porosity is already higher for DCPA-based tablets compared to MCC/mannitol or MCC/lactose. We also see slight changes in tensile strength, however these changes are more consistent at all storage conditions, unlike batches composed of MCC/mannitol or MCC/lactose, which show a more distinct difference depending on the storage humidity.

### 4.4 Effect of disintegrant choice

These results have primarily highlighted trends in the stability behaviour within each filler combination, as these are the most clear observations based on the data. However, disintegrant choice is also an integral part of the formulation selection. In this study, tablets containing XPVP tended to show the largest increases in porosity compared to tablets containing the same filler combination formulated with other disintegrants. This could be due to the increased moisture uptake capacity of XPVP (demonstrated by the moisture sorption data shown in Fig 1), leading to increased swelling during the disintegrant pre-activation. Aside from this observation, the change in other physical properties appears to be primarily driven by the fillers and disintegration mechanism.

## 5 Conclusions

This study investigated the effects of accelerated storage conditions on the physical properties of directly compressed placebo tablets. In terms of humidity, most formulations displayed increases in porosity and decrease in tensile strength after storage at high humidity. This can be explained by the premature expansion of swelling components (specifically MCC and disintegrants) as moisture is absorbed during storage. As a result, the microstructure of the tablet is permanently changed, even after removal from storage. The influence of storage temperature on the physical properties of tablets varied based on the formulation, however, the property most affected by storage was the initial contact angle. This suggests that high temperatures could affect the surface wettability of tablets during storage. For each batch studied, the initial performance-controlling mechanism could be considered to understand the changes observed after storage.

The focus of this study was to investigate the effects of different excipients and disintegration mechanisms on physical ageing. As such, uncoated tablets without an active pharmaceutical ingredient were used for this work. However, in a commercial setting most tablets would be coated during the manufacturing process. Tablet coating can offer several advantages, including improved product stability by protecting the tablet core from moisture. Having an understanding of the effects of storage on the core tablet components could inform the selection of coating and packaging materials.

It should also be noted that the scale of these changes in disintegration time (for example, a few minutes) would not generally be of concern in a commercial setting, and in fact, some of these changes (particularly those which occurred at the highest temperature and humidity conditions) may not occur during storage or stability studies performed under traditional ICH conditions. However, understanding the physical properties causing these changes can provide valuable mechanistic insight into the effects of these excipients and, consequently, provide a basis for excipient selection during formulation development. These physical changes could also have implications in a clinical setting, for example, to identify potential changes for patients who live in humid climates, or when mediation is stored in bathroom cabinets where moisture absorption is likely. Finally, future studies with additional timepoints within the first 2 weeks of the study would provide more information on the shape of the stability profiles for each property, and therefore facilitate modelling of these changes.

## Declaration of Competing Interest

The authors declare the following financial interests/personal relationships which may be considered as potential competing interests:

Ibrahim Khadra reports financial support was provided by 10.13039/501100000266Engineering and Physical Sciences Research Council. Ibrahim Khadra reports financial support was provided by 10.13039/100004325AstraZeneca PLC. Daniel Markl reports a relationship with AstraZeneca PLC that includes: funding grants and non-financial support. Ibrahim Khadra reports a relationship with AstraZeneca PLC that includes: funding grants and non-financial support. Natalie Maclean reports a relationship with AstraZeneca PLC that includes: non-financial support. James Mann reports a relationship with AstraZeneca PLC that includes: employment. Helen Williams reports a relationship with AstraZeneca PLC that includes: employment. Alexander Abbott reports a relationship with AstraZeneca PLC that includes: employment. Heather Mead reports a relationship with AstraZeneca PLC that includes: employment. NA

## Appendix A Supplementary data

Supporting Information Image 1
