# Supplementary material for: Investigating the role of excipients on the physical stability of directly compressed tablets
Source: Int J Pharm X. 2021 Dec 9;4:100106. doi: 10.1016/j.ijpx.2021.100106 (PMC8688555; doi:10.1016/j.ijpx.2021.100106)
Supplement: Supplementary file 1 — Supporting Information [file mmc1.pdf]

Supporting Information to

Investigating the Role of Excipients on the Physical Stability of Directly Compressed Tablets

Natalie Maclean[a], Ibrahim Khadra[a], James Mann[b], Helen Williams[c], Alexander Abbott[b], Heather Mead[c], Daniel Markl[a,d,*]

[a]*Strathclyde Institute of Pharmacy & Biomedical Sciences, University of Strathclyde, Glasgow, UK*
[b]*Oral Product Development, Pharmaceutical Technology & Development, Operations, AstraZeneca, Macclesfield, UK*
[c]*New Modalities and Parenteral Development, Pharmaceutical Technology & Development, Operations, AstraZeneca, Macclesfield, UK*
[d]*Future Continuous Manufacturing and Advanced Crystallisation (CMAC) Research Hub, University of Strathclyde, Glasgow, UK*

Abstract

This supporting information provides further stability data collected during the analysis of the placebo batches.

*Corresponding Author: daniel.markl@strath.ac.uk

Tensile Strength

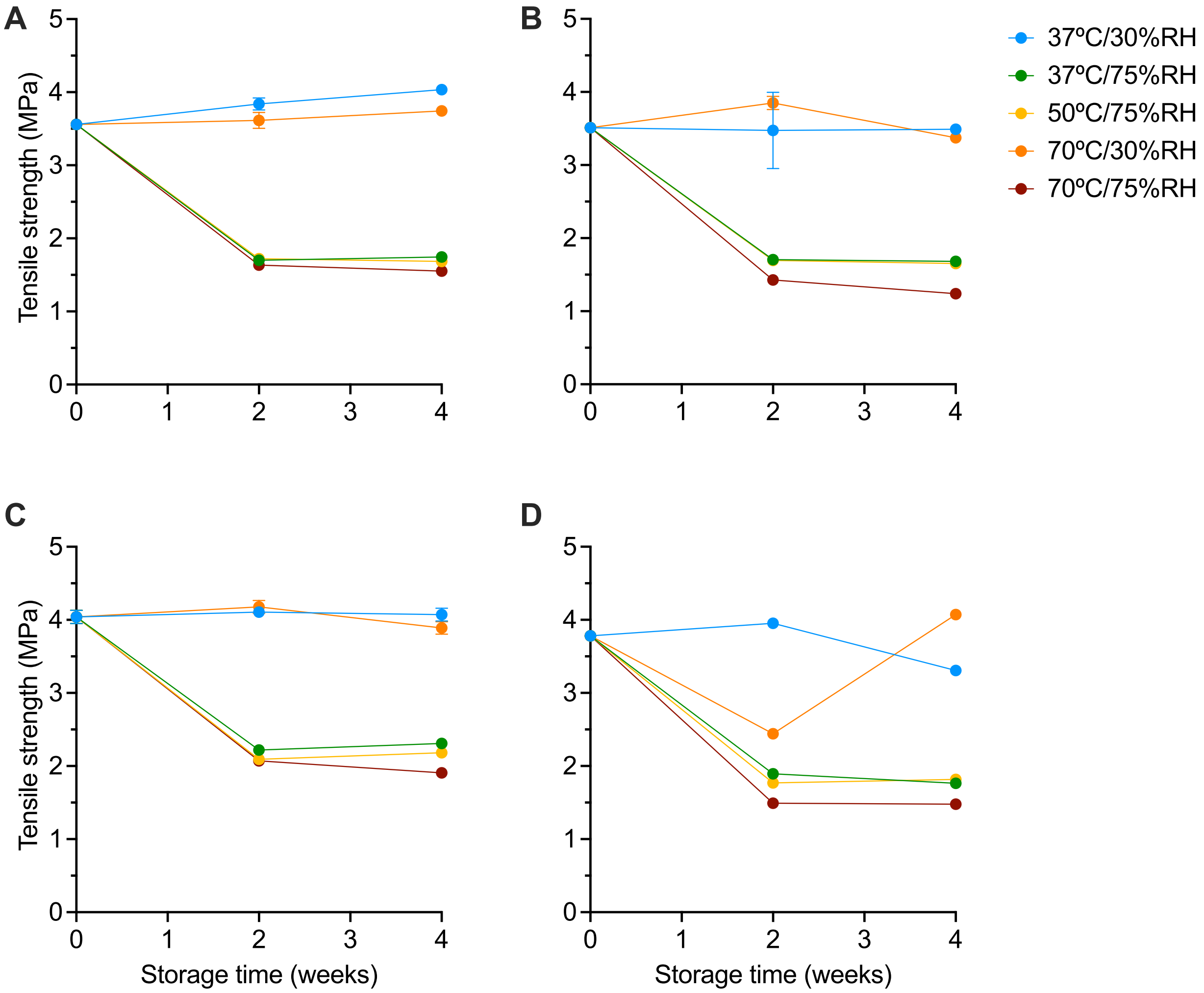

Figure S1: The change in tensile strength for MCC/lactose-based tablets with (A) CCS, (B) XPVP, (C) L-HPC and (D) SSG after storage under accelerated storage conditions for 2 and 4 weeks. Mean ± standard deviation, n = 10.

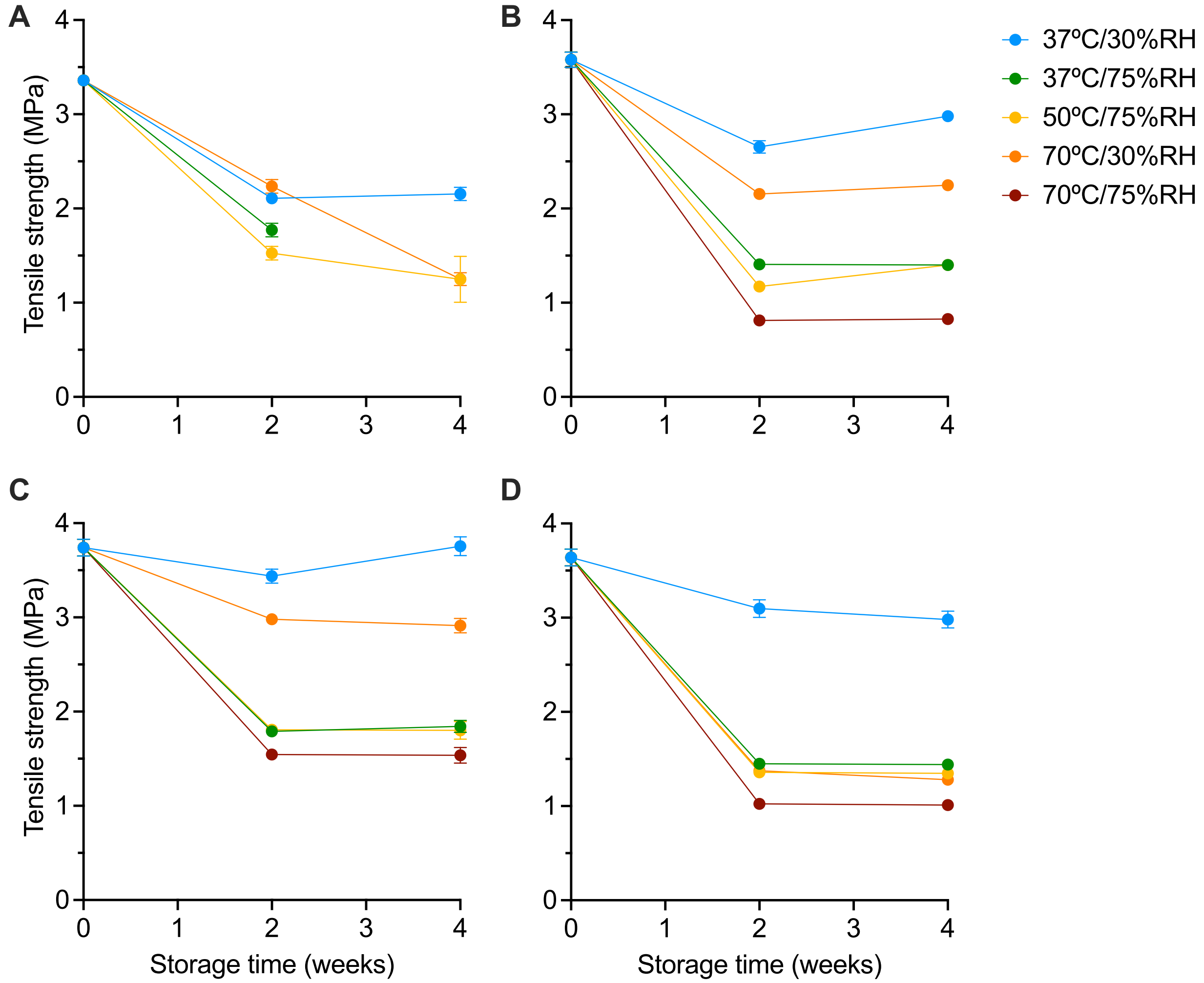

Figure S2: The change in tensile strength for MCC/mannitol-based tablets with (A) CCS, (B) XPVP, (C) L-HPC and (D) SSG after storage under accelerated storage conditions for 2 and 4 weeks. Mean ± standard deviation, n = 10.

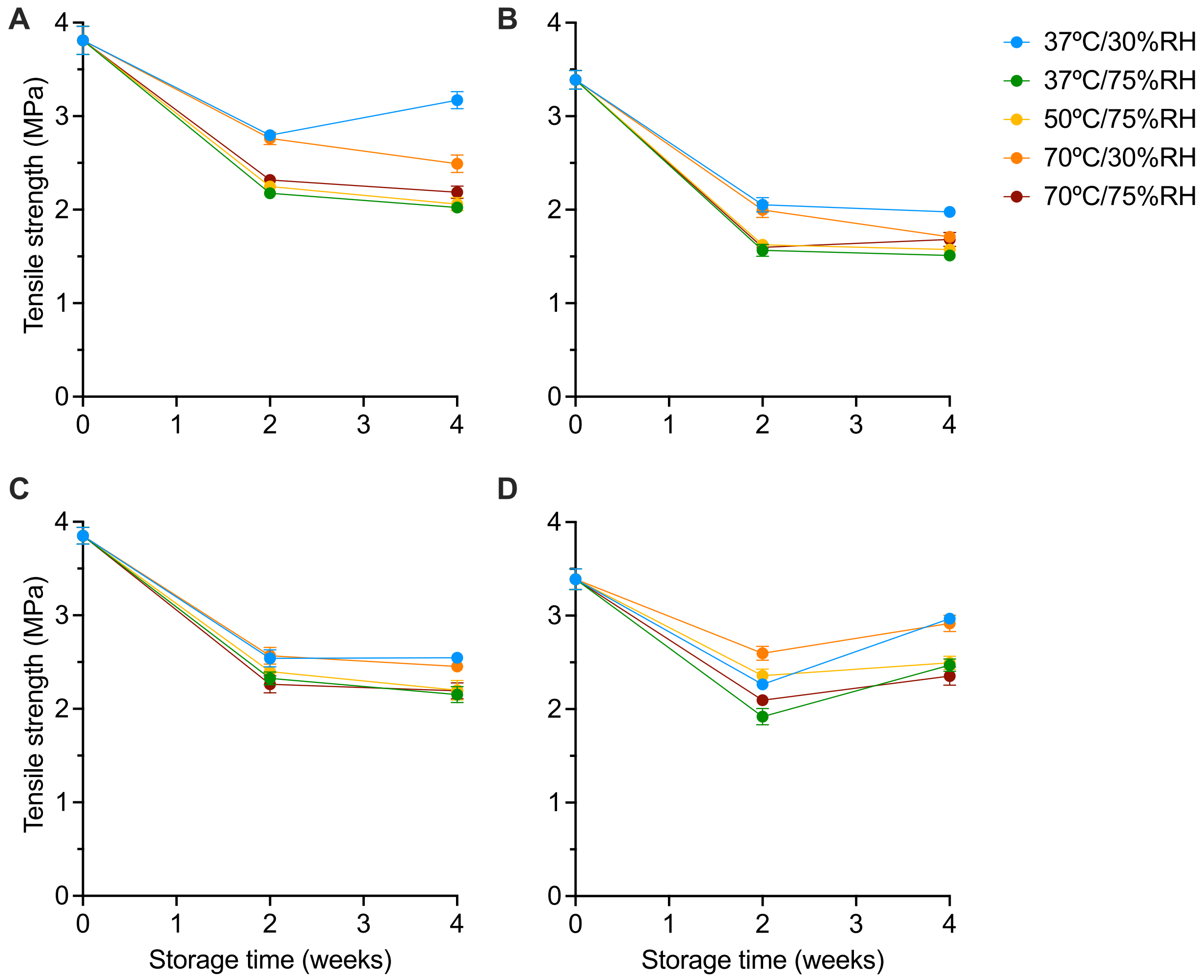

Figure S3: The change in tensile strength for MCC/DCPA-based tablets with (A) CCS, (B) XPVP, (C) L-HPC and (D) SSG after storage under accelerated storage conditions for 2 and 4 weeks. Mean ± standard deviation, n = 10.

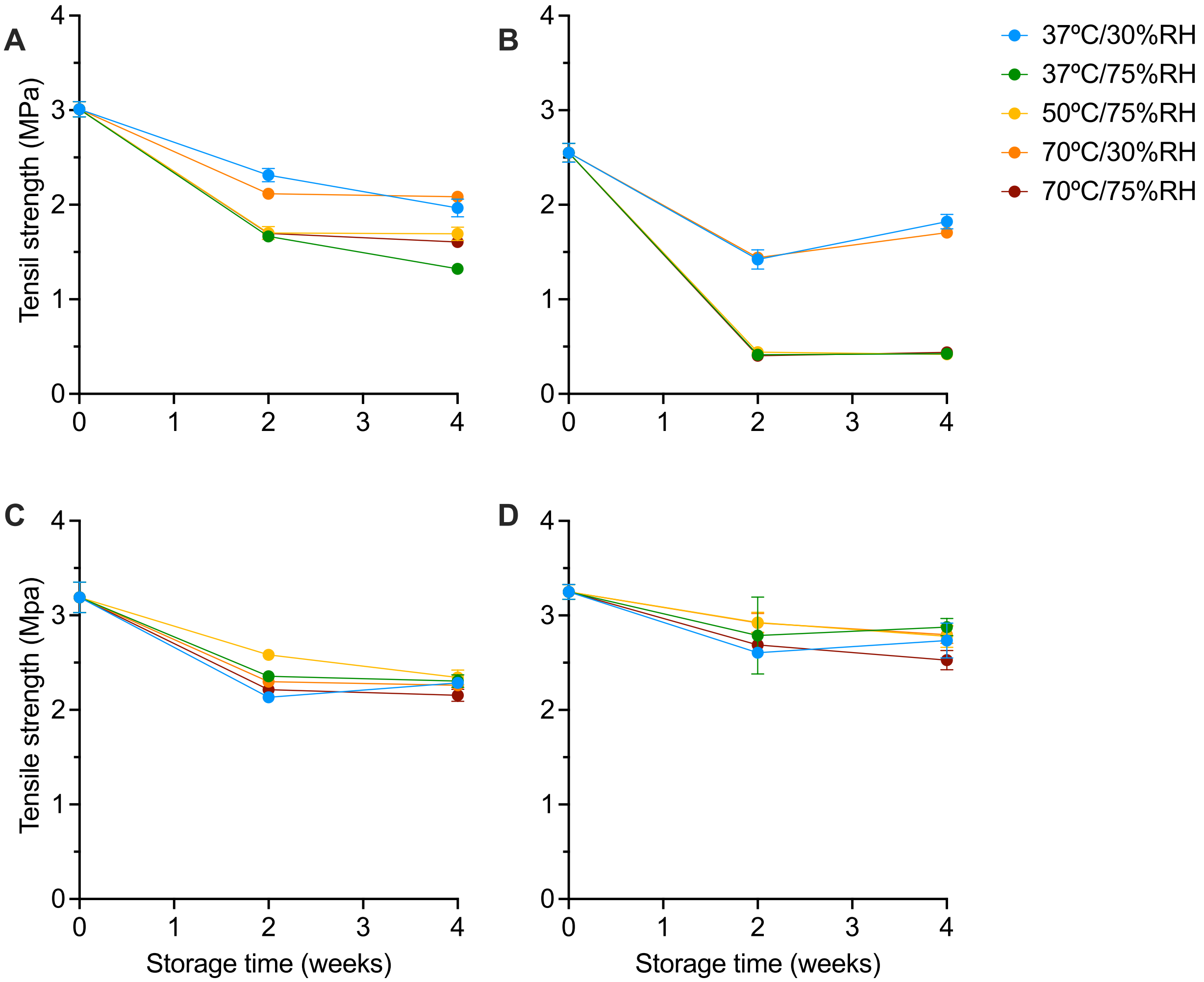

Figure S4: The change in tensile strength for DCPA/lactose-based tablets with (A) CCS, (B) XPVP, (C) L-HPC and (D) SSG after storage under accelerated storage conditions for 2 and 4 weeks. Mean ± standard deviation, n = 10.

Porosity

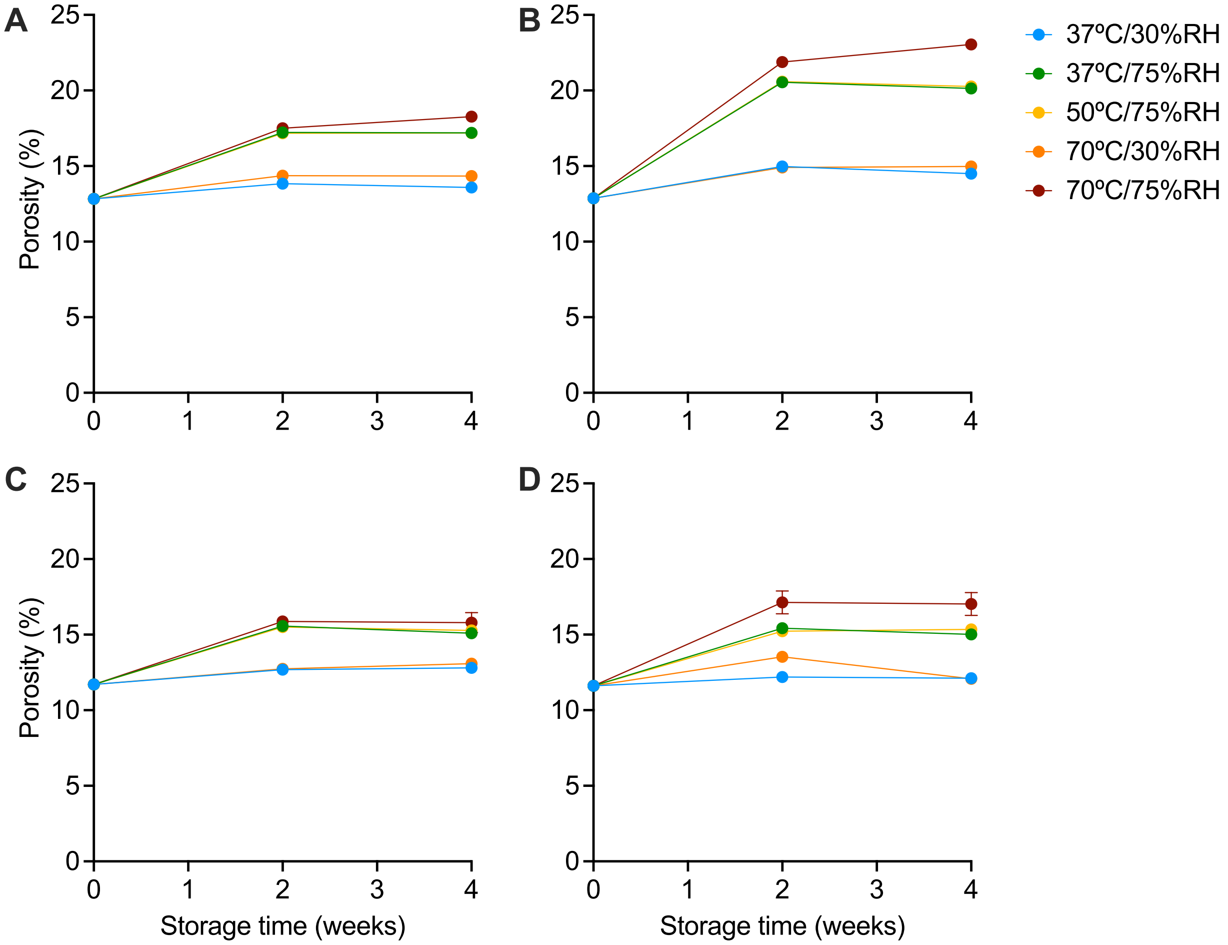

Figure S5: The change in porosity for MCC/lactose-based tablets with (A) CCS, (B) XPVP, (C) L-HPC and (D) SSG after storage under accelerated storage conditions for 2 and 4 weeks. Mean ± standard deviation, n = 100 (0 weeks) and n = 10 (2 and 4 weeks).

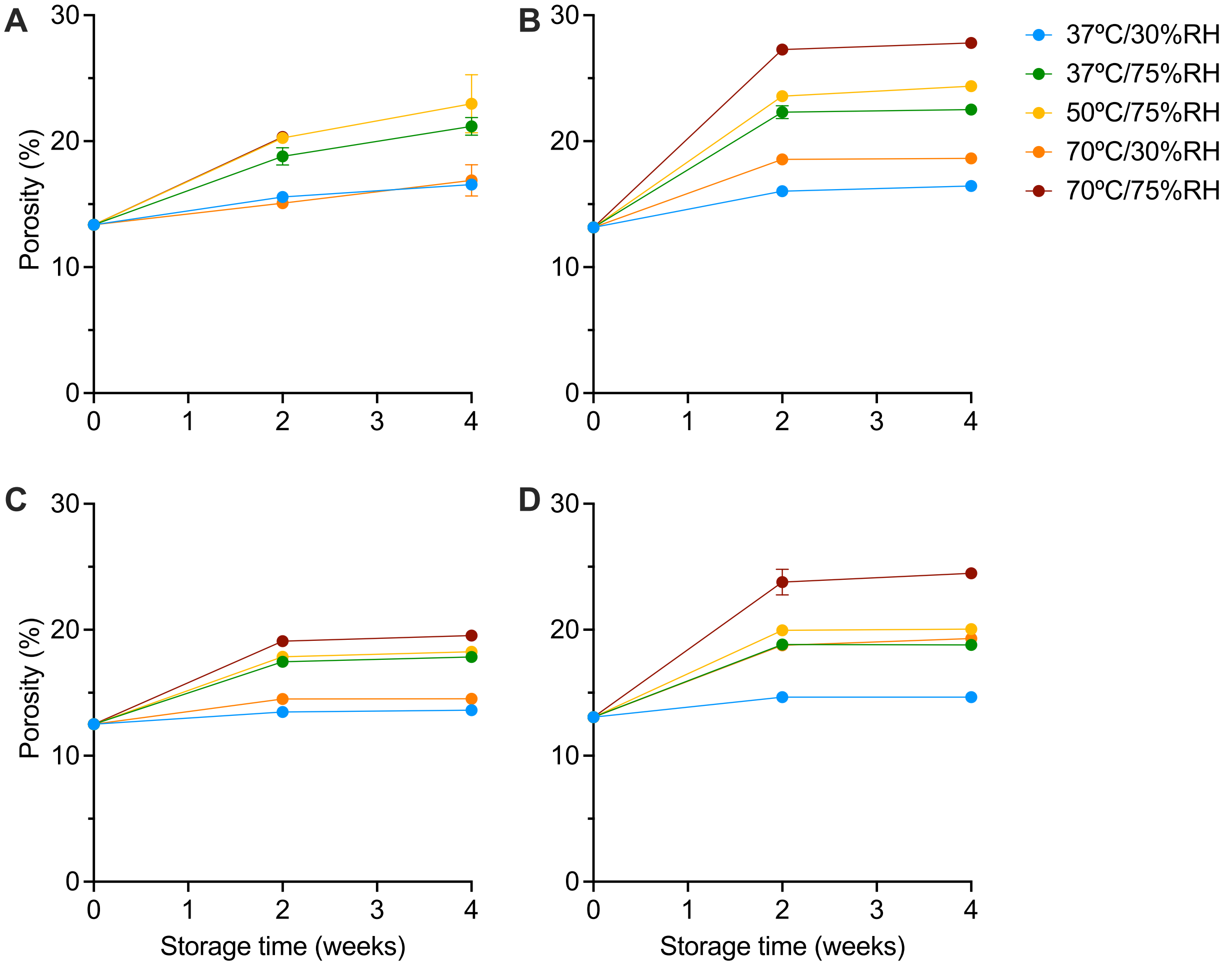

Figure S6: The change in porosity for MCC/mannitol-based tablets with (A) CCS, (B) XPVP, (C) L-HPC and (D) SSG after storage under accelerated storage conditions for 2 and 4 weeks. Mean ± standard deviation, n = 100 (0 weeks) and n = 10 (2 and 4 weeks).

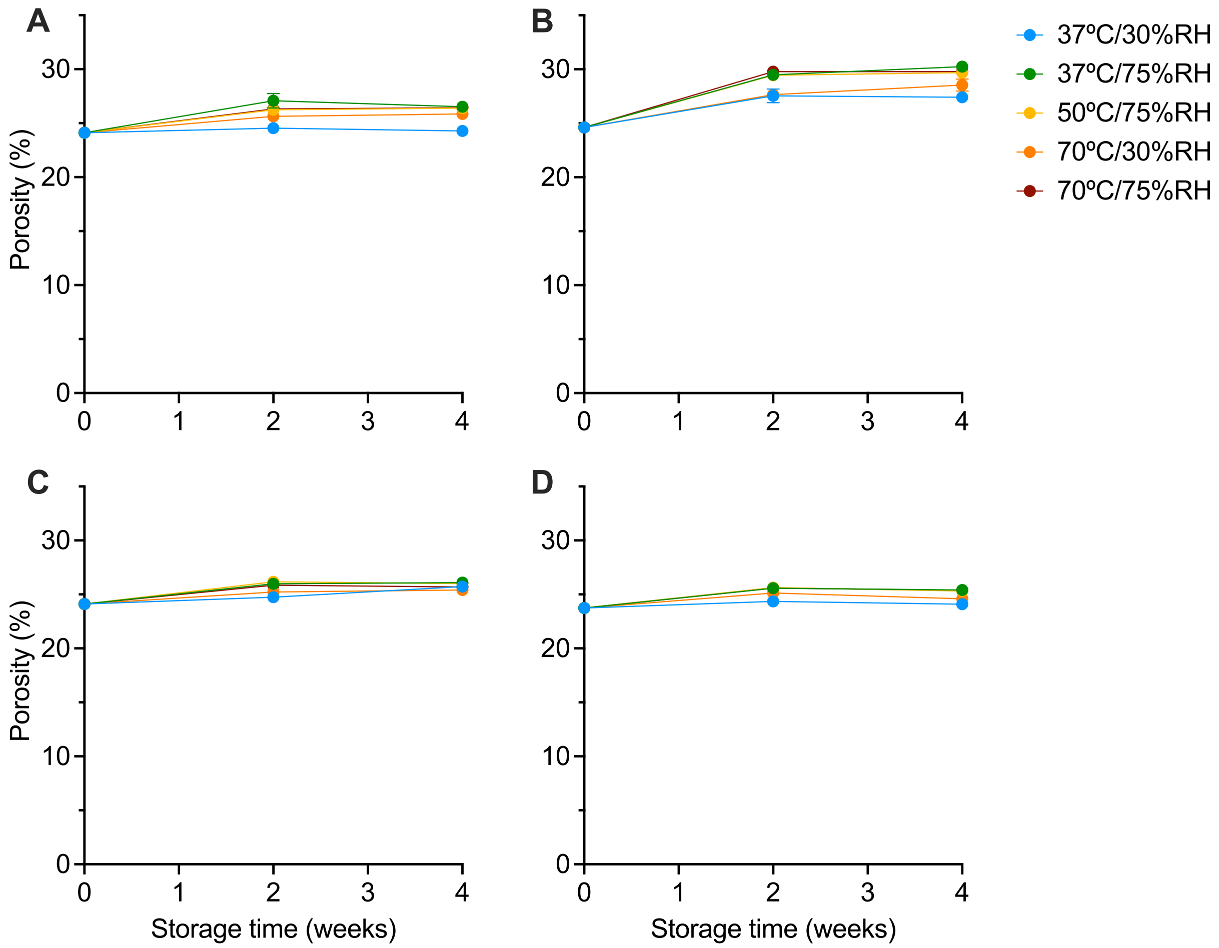

Figure S7: The change in porosity for MCC/DCPA-based tablets with (A) CCS, (B) XPVP, (C) L-HPC and (D) SSG after storage under accelerated storage conditions for 2 and 4 weeks. Mean ± standard deviation, n = 100 (0 weeks) and n = 10 (2 and 4 weeks).

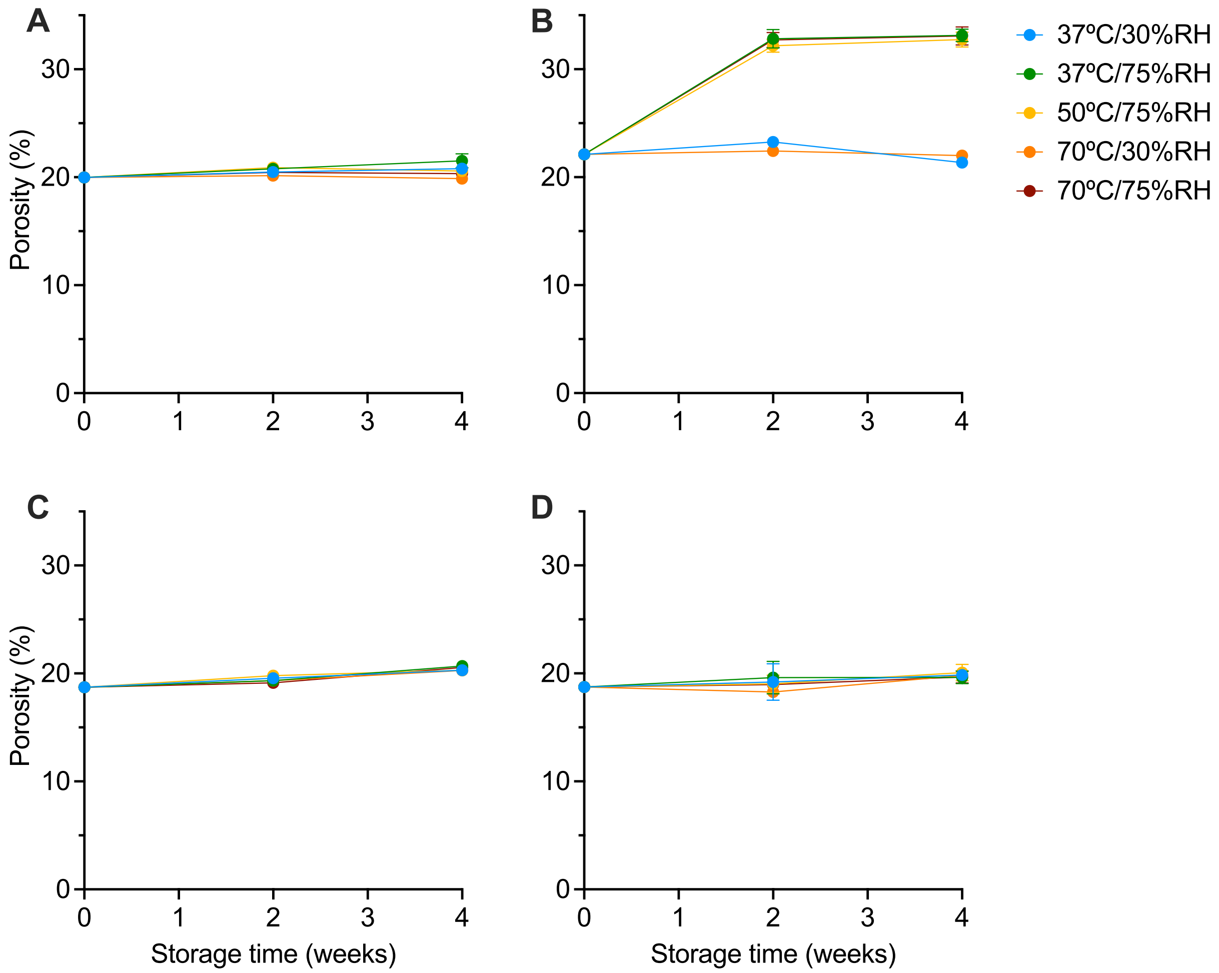

Figure S8: The change in porosity for DCPA/lactose-based tablets with (A) CCS, (B) XPVP, (C) L-HPC and (D) SSG after storage under accelerated storage conditions for 2 and 4 weeks. Mean ± standard deviation, n = 100 (0 weeks) and n = 10 (2 and 4 weeks).

Contact Angle

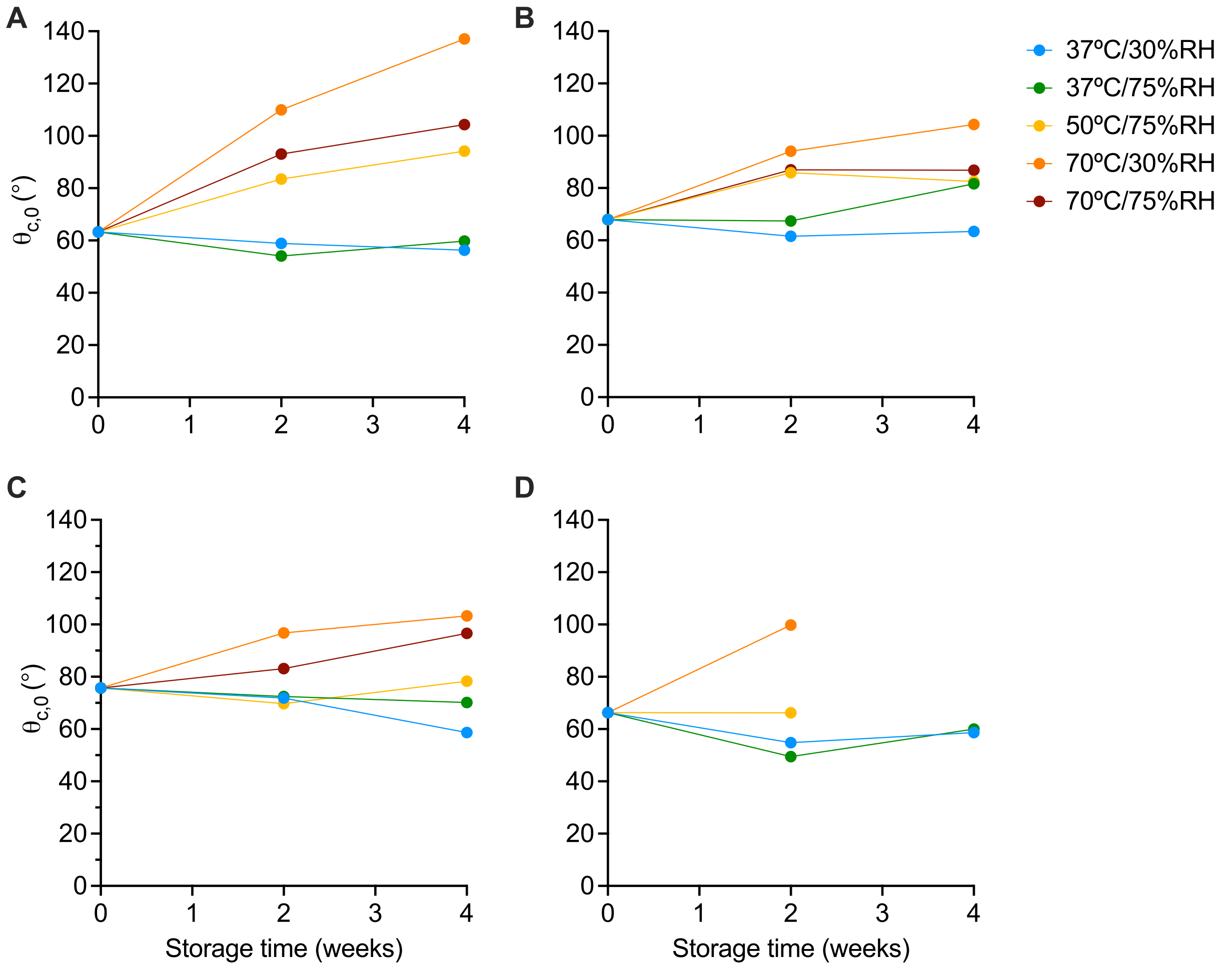

Figure S9: The change in initial contact angle ($\theta_{c,0}$) for MCC/lactose-based tablets with (A) CCS, (B) XPVP, (C) L-HPC and (D) SSG after storage under accelerated storage conditions for 2 and 4 weeks. Mean, n = 2.

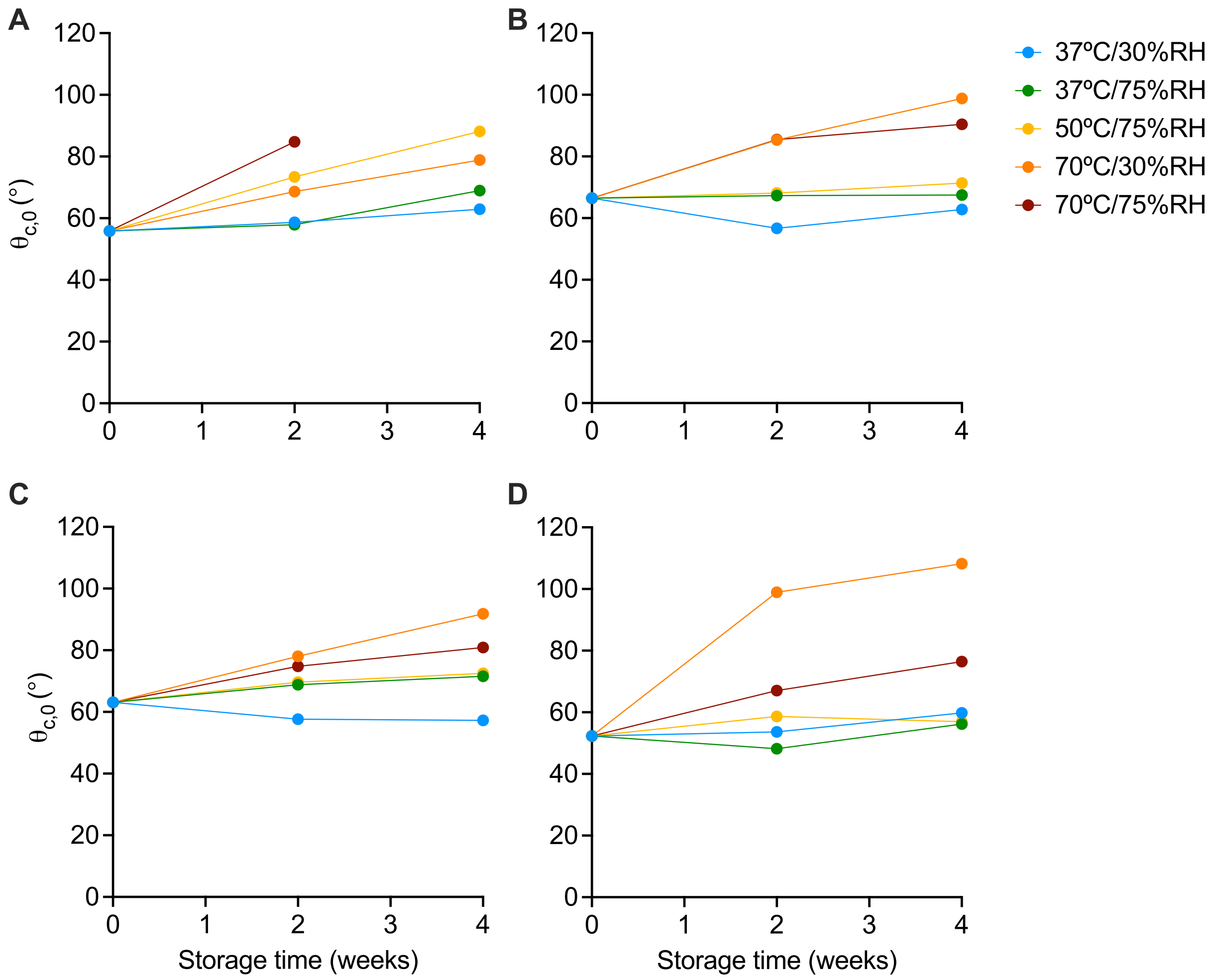

Figure S10: The change in initial contact angle ($\theta_{c,0}$) for MCC/mannitol-based tablets with (A) CCS, (B) XPVP, (C) L-HPC and (D) SSG after storage under accelerated storage conditions for 2 and 4 weeks. Mean, n = 2.

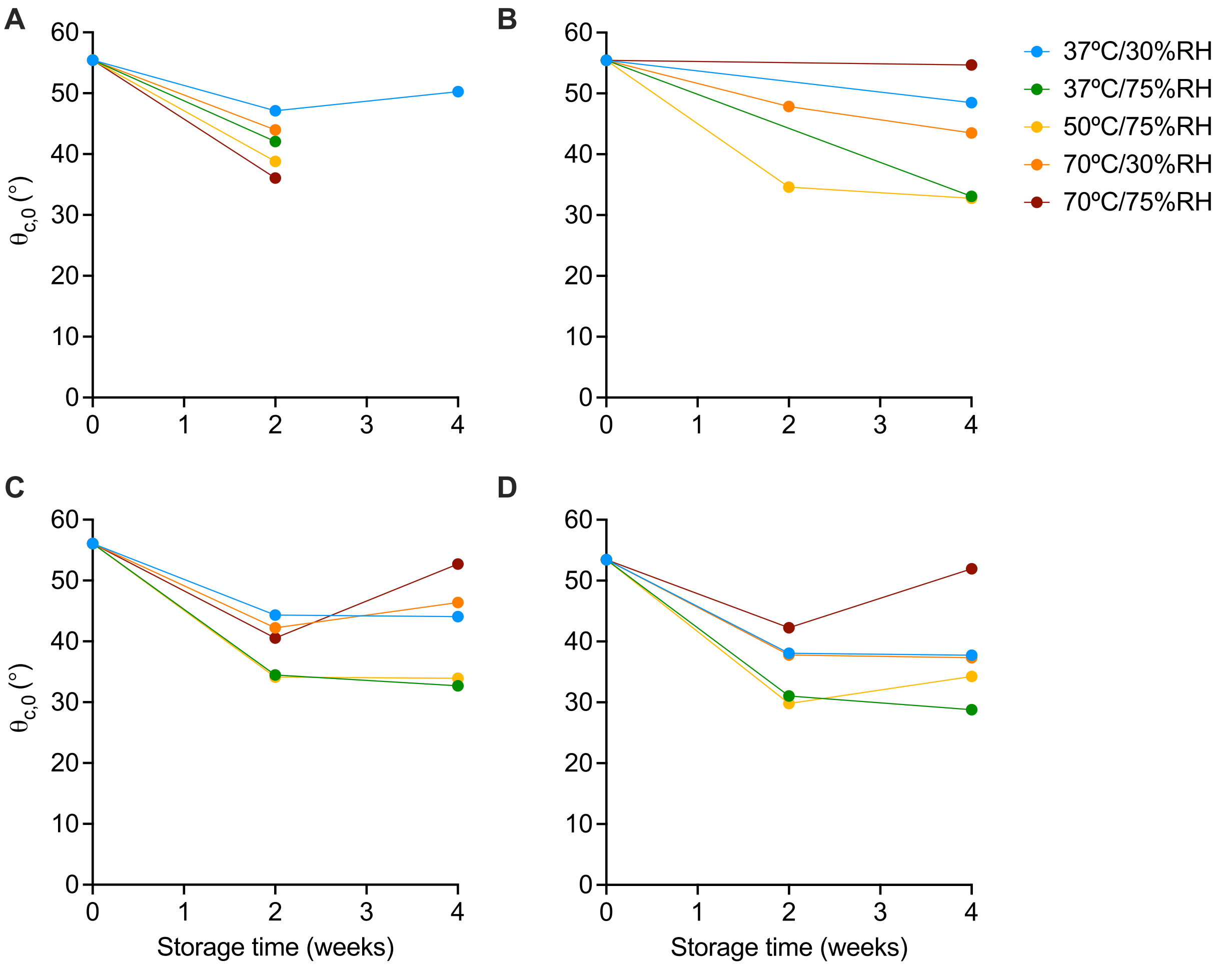

Figure S11: The change in initial contact angle ($\theta_{c,0}$) for MCC/DCPA-based tablets with (A) CCS, (B) XPVP, (C) L-HPC and (D) SSG after storage under accelerated storage conditions for 2 and 4 weeks. Mean, n = 2.

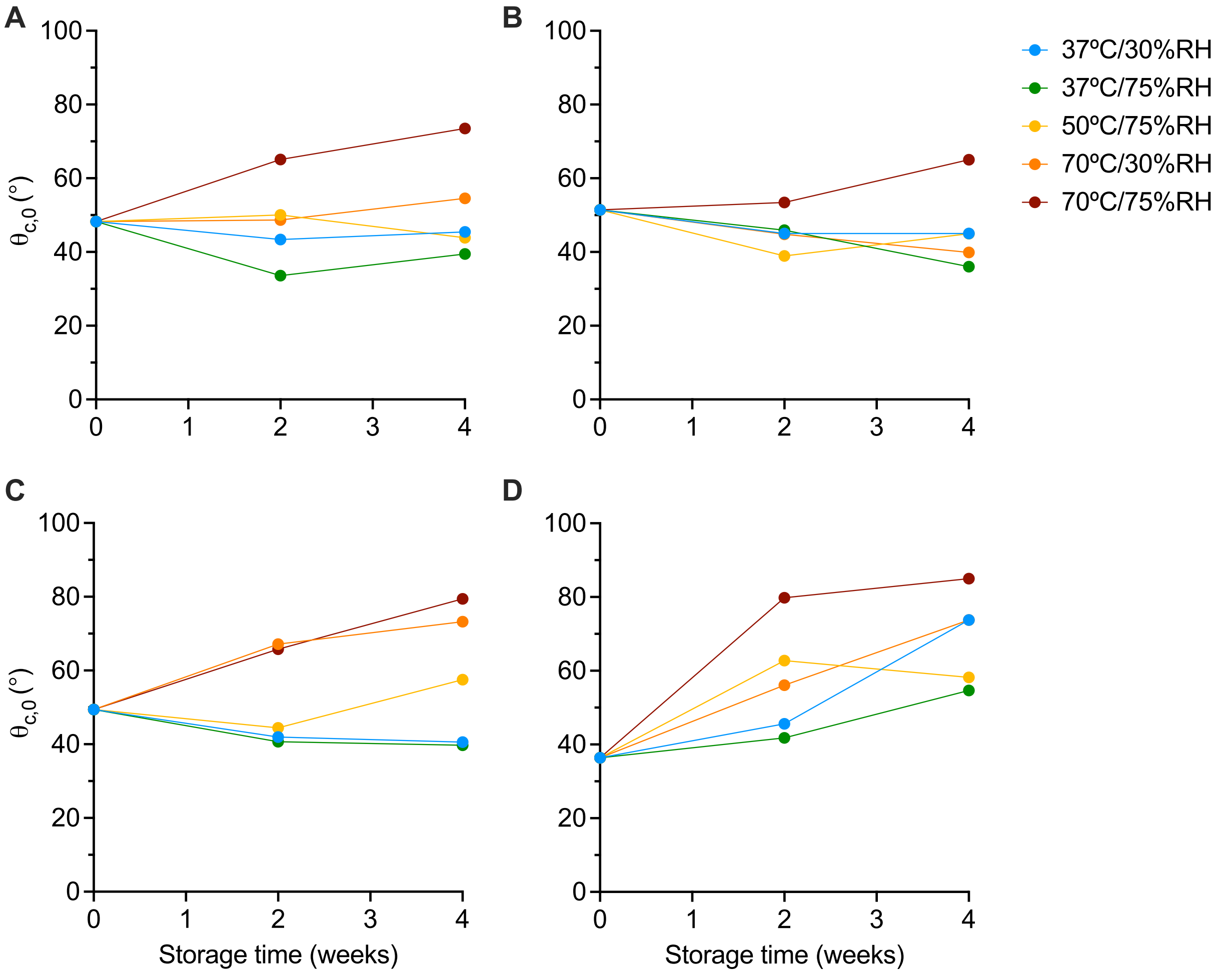

Figure S12: The change in initial contact angle ($\theta_{c,0}$) for DCPA/lactose-based tablets with (A) CCS, (B) XPVP, (C) L-HPC and (D) SSG after storage under accelerated storage conditions for 2 and 4 weeks. Mean, n = 2.

Disintegration Time

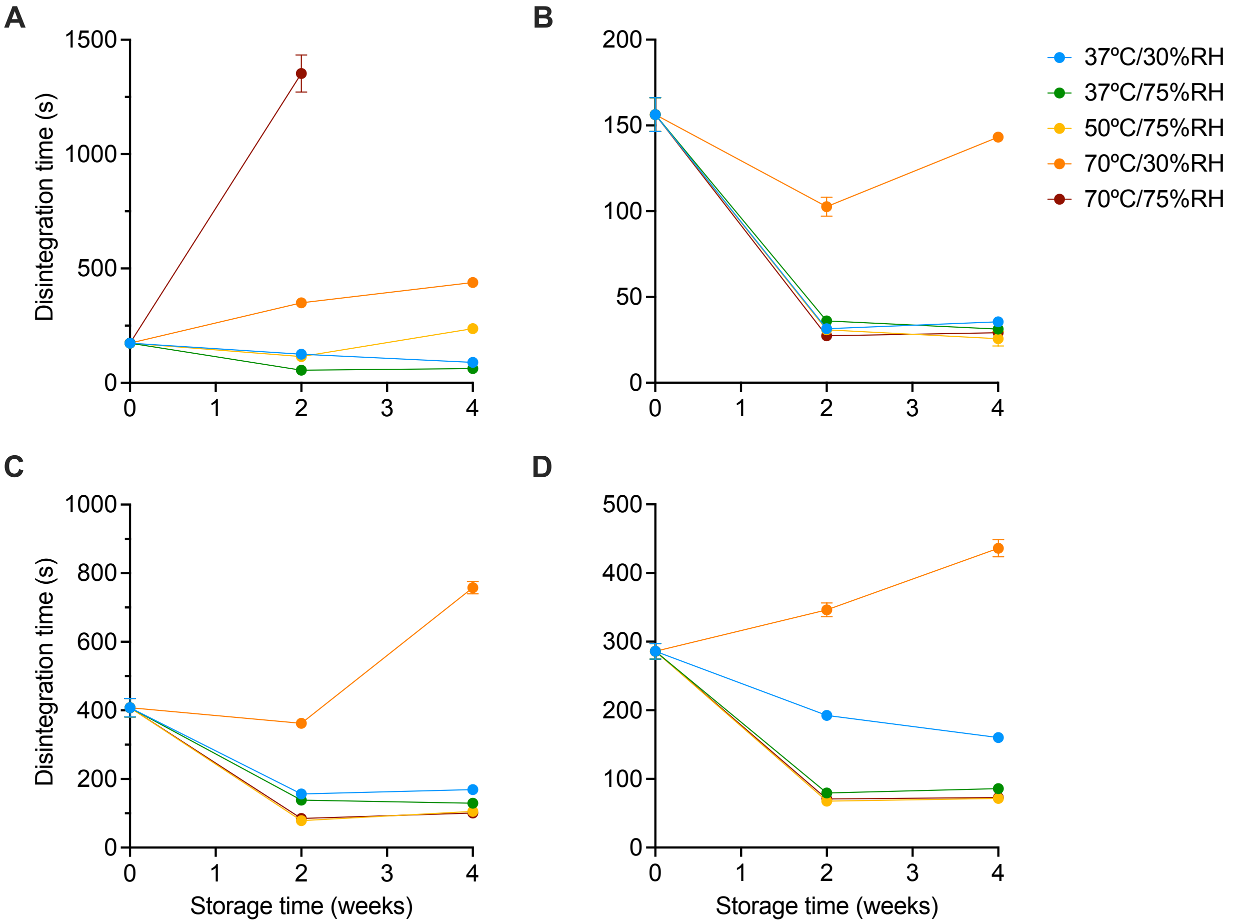

Figure S13: The change in disintegration time for MCC/lactose-based tablets with (A) CCS, (B) XPVP, (C) L-HPC and (D) SSG after storage under accelerated storage conditions for 2 and 4 weeks. Mean ± standard deviation, n = 6.

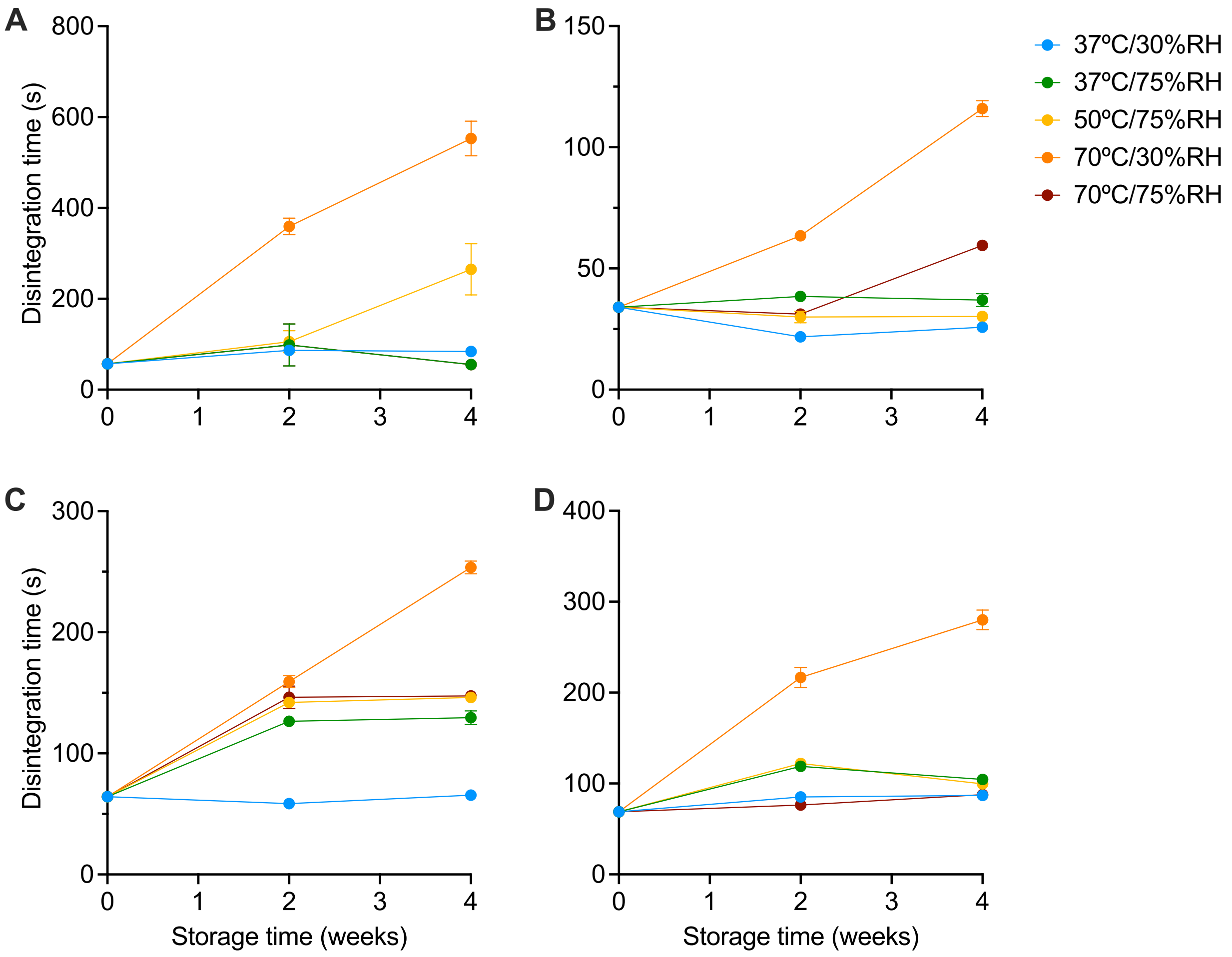

Figure S14: The change in disintegration time for MCC/mannitol-based tablets with (A) CCS, (B) XPVP, (C) L-HPC and (D) SSG after storage under accelerated storage conditions for 2 and 4 weeks. Mean ± standard deviation, n = 6.

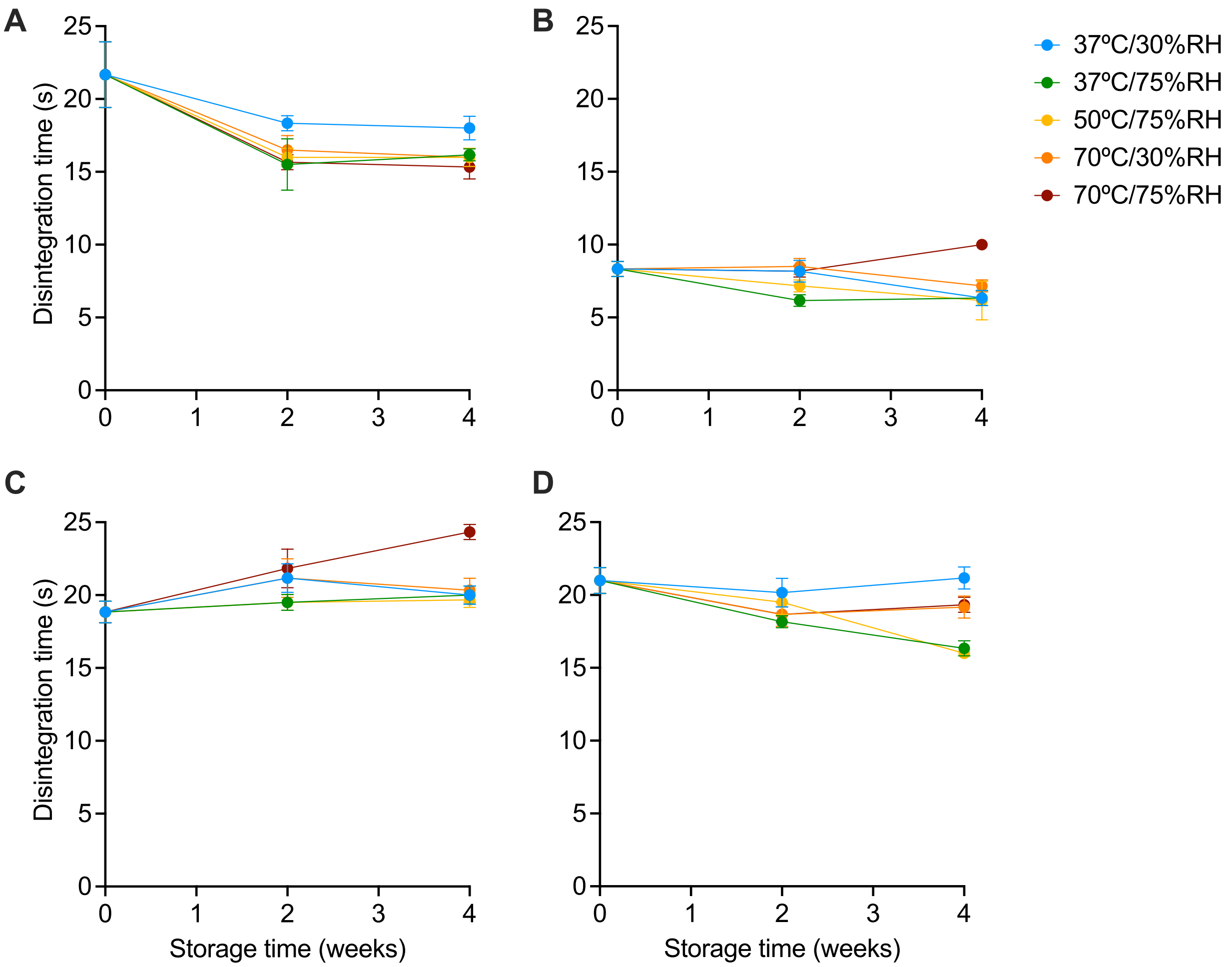

Figure S15: The change in disintegration time for MCC/DCPA-based tablets with (A) CCS, (B) XPVP, (C) L-HPC and (D) SSG after storage under accelerated storage conditions for 2 and 4 weeks. Mean ± standard deviation, n = 6.

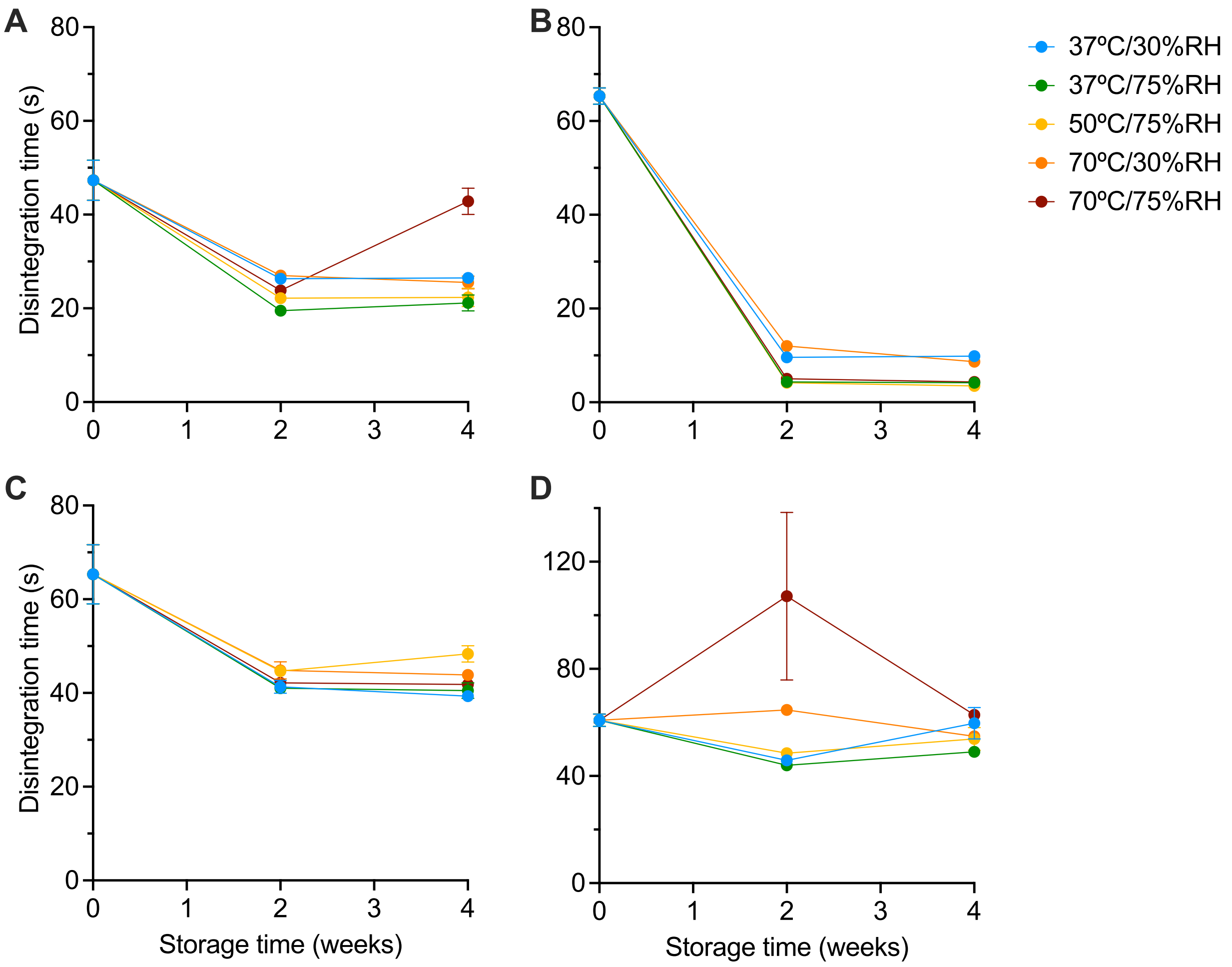

Figure S16: The change in disintegration time for DCPA/lactose-based tablets with (A) CCS, (B) XPVP, (C) L-HPC and (D) SSG after storage under accelerated storage conditions for 2 and 4 weeks. Mean ± standard deviation, n = 6.
